# Small RNA ArcZ Regulates Oxidative Stress Response Genes and Regulons in *Erwinia amylovora*

**DOI:** 10.3389/fmicb.2019.02775

**Published:** 2019-11-29

**Authors:** Jeffrey K. Schachterle, Daphne M. Onsay, George W. Sundin

**Affiliations:** ^1^Genetics Graduate Program, Michigan State University, East Lansing, MI, United States; ^2^Department of Plant, Soil, and Microbial Sciences, Michigan State University, East Lansing, MI, United States

**Keywords:** sRNA, ArcZ, catalase, fire blight, *Erwinia amylovora*, peroxide

## Abstract

*Erwinia amylovora*, causative agent of fire blight disease of apple and pear trees, has evolved to use small RNAs for post-transcriptional regulation of virulence traits important for disease development. The sRNA ArcZ regulates several virulence traits, and to better understand its roles, we conducted a transcriptomic comparison of wild-type and Δ*arcZ* mutant *E. amylovora*. We found that ArcZ regulates multiple cellular processes including genes encoding enzymes involved in mitigating the threat of reactive oxygen species (*katA*, *tpx*, *osmC*), and that the Δ*arcZ* mutant has reduced catalase activity and is more susceptible to exogenous hydrogen peroxide. We quantified hydrogen peroxide production by apple leaves inoculated with *E. amylovora* and found that the while wild-type *E. amylovora* cells produce enough catalase to cope with defense peroxide, the Δ*arcZ* mutant is likely limited in virulence because of inability to cope with peroxide levels in host leaves. We further found that the ArcZ regulon overlaps significantly with the regulons of transcription factors involved in oxidative sensing including Fnr and ArcA. In addition, we show that ArcZ regulates *arcA* at the post-transcriptional level suggesting a role for this system in mediating adaptations to oxidative state, especially during disease development.

## Introduction

When pathogenic microbes arrive on a host plant, the plant perceives the arrival of a threat through recognition of pathogen associated molecular patterns (PAMPs) (Jones and Dangl, 2006). The recognized patterns include conserved molecules associated with pathogenic microbes, such as chitin (Miya et al., 2007), flagellin (Chinchilla et al., 2007), and translation elongation factor Tu (Kunze et al., 2004). The binding of these PAMPs to surface receptors triggers a complex signaling cascade that activates defense responses (Jones and Dangl, 2006). Host plant defense responses are diverse and include actions such as stomatal closure (Melotto et al., 2006), hormone signaling (Heese et al., 2007), callose deposition (Thilmony et al., 2006), and production of reactive oxygen species (Bolwell and Wojtaszek, 1997). Plant pathogenic microbes have responded to these host defenses through the evolution of effector proteins that act to suppress and subvert host defense signaling and activity (Guo et al., 2009). In the case of bacterial pathogens, the effectors are often translocated directly into the host cytoplasm via the type III secretion system, a needle-like protein structure (He, 1998). In an ongoing biochemical arms race, hosts and pathogens alike have evolved numerous effector-target relationships that affect disease outcomes (Jones and Dangl, 2006; Asai and Shirasu, 2015; Khan et al., 2016). For many bacterial pathogens, this has resulted in a number of effector proteins that are essential for full virulence (Jamir et al., 2004; Toruño et al., 2016). In addition to effector proteins, bacterial pathogens have evolved additional virulence strategies that allow them to flourish in the environment of a host plant and avoid host defenses. For example, *Erwinia amylovora*, causative agent of fire blight disease of apple and pear trees, utilizes several virulence strategies to avoid, suppress, and cope with host defenses (Geier and Geider, 1993; Bogdanove et al., 1998; Berry et al., 2009; Koczan et al., 2009, 2011; Zhao et al., 2010). For pathogenesis, *E. amylovora* requires effective translocation of the type III effectors DspE and AvrRpt2_Ea_ into host cells to suppress host defenses and induce necrosis (Bogdanove et al., 1998; Zhao et al., 2006). Additional virulence traits that play a key role for *E. amylovora* include exopolysaccharide production and biofilm formation (Bellemann et al., 1994; Nimtz et al., 1996; Koczan et al., 2009; Castiblanco and Sundin, 2018), motility (Raymundo and Ries, 1980; Bayot and Ries, 1986), ability to mitigate the threat of reactive oxygen species (Santander et al., 2018), and ability to acquire and utilize essential nutrients (Aldridge et al., 1997; Dellagi et al., 1998).

Production of the exopolysaccharides amylovoran (Bellemann et al., 1994; Nimtz et al., 1996), levan (Gross et al., 1992; Koczan et al., 2009), and cellulose (Castiblanco and Sundin, 2018) along with proteinaceous attachment structures (Koczan et al., 2011) contribute to biofilm formation. Biofilm formation provides protective layers that can serve to both prevent host defense molecules, like reactive oxygen species, from reaching the bacteria (Danhorn and Fuqua, 2007), and to conceal the bacteria from host detection, reducing the degree of host defense response (D’Haeze and Holsters, 2004). Motility enables bacteria to use flagella or pili to migrate and move to more favorable locations where host defenses may be reduced or nutrient availability may be more favorable (Bayot and Ries, 1986; Berry et al., 2009). Although *E. amylovora* can be concealed through some virulence traits, move away from host defenses, and even directly reduce the host defense response through type III effectors, the bacteria will still have to cope with host defense compounds and responses as well as acquire sufficient nutrients to maintain growth during infection (Kamber et al., 2016). Thus, the ability to face host defenses and mitigate the threat of reactive oxygen species is also critical for full virulence (Santander et al., 2018). To coordinately express each virulence-associated trait under the precise conditions, *E. amylovora* has evolved elaborate environmental sensing and signal transduction cascades (Zhao et al., 2009). Efforts to characterize these regulatory pathways have successfully linked several regulatory systems with virulence associated traits.

Recent work has revealed the importance of small non-coding RNAs (sRNAs) in the regulation of virulence and virulence-associated traits in *E. amylovora* (Zeng et al., 2013; Zeng and Sundin, 2014). sRNAs are typically involved in post-transcriptional regulation. One class of sRNAs that affects virulence in *E. amylovora* includes those that are dependent on the chaperone protein Hfq (Zeng et al., 2013). The Hfq chaperone stabilizes a family of *trans-*acting sRNAs that regulate targets by RNA-RNA base-pairing (Sun et al., 2002; Vogel and Luisi, 2011; Sauer et al., 2012). In *E. amylovora*, 42 Hfq-dependent sRNAs have been identified, and the Hfq-dependent sRNA ArcZ in particular is critical for virulence and several virulence-associated traits including production of the exopolysaccharides levan and amylovoran, normal biofilm formation, flagellar motility and translocation of type III effectors to plant cells (Zeng and Sundin, 2014). We have recently shown that ArcZ regulates flagellar motility in *E. amylovora* through a direct interaction with the flagellar master regulator FlhD (Schachterle et al., 2019) and that ArcZ impacts exopolysaccharide production and biofilm formation through the leucine responsive regulator protein Lrp (Schachterle and Sundin, 2019). However, it is not known if there are further virulence-associated traits being regulated by ArcZ, nor is it known how ArcZ regulates type III secretion.

Because of the breadth of phenotypes ArcZ regulates, we conducted a transcriptomic comparison of the Δ*arcZ* mutant relative to wild-type to gain additional insights into the mechanisms of ArcZ regulation of virulence-associated traits. In addition to previously known interactions between ArcZ and Lrp, we found that ArcZ regulates several genes involved in mitigating the threat of reactive oxygen species, and present evidence that this regulation is critical for *in planta* survival. We also found a significant amount of overlap between the ArcZ regulon and regulons of global transcription factors associated with oxidative state signaling, including the ArcBA (anoxic redox control) two-component system. We further present evidence that ArcZ regulates *arcA* post-transcriptionally, indicating that ArcZ plays a major role in the oxidative status responsive regulatory pathways.

## Materials and Methods

### Strain Growth and Culture Conditions

Bacterial strains were routinely grown using LB culture media. *E. amylovora* strains were cultured at 28°C and *Escherichia coli* strains were cultured at 37°C. When appropriate, antibiotics were used in the following final concentrations: ampicillin 100 μg mL^–1^, kanamycin 30 μg mL^–1^, chloramphenicol 20 μg mL^–1^. Bacterial strains and oligonucleotides used in this study are found in Table 1 and Supplementary Table S1, respectively.

**TABLE 1:** Bacterial strains and plasmids used in this work.

**Strains and plasmids**	**Relevant characteristics**	**Source or reference**
***Escherichia coli***		
DH5α		Invitrogen
***Erwinia amylovora***		
Ea1189	Wild-type	GSPB^a^
Ea1189 Δ*arcZ*	*arcZ* deletion mutant	Zeng et al., 2013
Ea1189 Δ*katA*	*katA* deletion mutant	This work
Ea1189 Δ*katG*	*katG* deletion mutant	This work
Ea1189 Δ*tpx*	*tpx* deletion mutant	This work
Ea1189 Δ*osmC*	*osmC* deletion mutant	This work
Ea1189 Δ*arcA*	*arcA* deletion mutant	This work
Ea1189 Δ*arcB*	*arcB* deletion mutant	This work
Ea1189 Δ*fnr*	*fnr* deletion mutant	This work
Ea1189 Δ*fur*	*fur* deletion mutant	This work
**Plasmids**		
pML-ArcZ	*arcZ* complementation	Zeng et al., 2013
pHM-tac:ArcZ	*arcZ* Over-expression, IPTG inducible tac promoter	Schachterle et al., 2019
pBBR1:katA	*katA* complementation	This work
pBBR1:katG	*katG* complementation	This work
pBBR1:tpx	*tpx* complementation	This work
pBBR1:osmC	*osmC* complementation	This work
pXG20-KatA	*katA* translational fusion	This work
pPROBE-KatA	*katA* promoter fusion	This work
pXG20-Tpx	*tpx* translational fusion	This work
pXG20-ArcA	*arcA* translational fusion	This work
pXG20-ArcB	*arcB* translational fusion	This work
pXG20-Fur	*fur* translational fusion	This work

*^*a*^GSPB, Göttinger Sammlung phytopathogener Bakterien, Göttingen, Germany.*

### RNA Extraction and Sequencing

RNA was isolated from cells induced in *hrp-*inducing minimal medium (HIMM, Huynh et al., 1989). For induction, cells were grown overnight in one volume of LB medium, collected by centrifugation, washed with HIMM, and then resuspended in an equal volume of HIMM and incubated at 28°C with shaking for the time specified. RNA was extracted using the approach of Rivas et al. (2001), with documented specific modifications (Schachterle and Sundin, 2019). RNA was quantified using the Qubit fluorescence method (Thermo Fisher Scientific, Waltham, MA, United States). RNA quality was ensured by visualization of ribosomal RNA bands in agarose gel and by LabChipGX HS RNA analysis (Caliper Life Sciences, Waltham, MA, United States). Total RNA was depleted of ribosomal RNA using bacterial Ribo-Zero kits (Illumina, San Diego, CA, United States) and remaining RNA was used for library preparation with the Illumina TruSeq Stranded Total RNA Library Preparation Kit on a Perkin Elmer Sciclone G3 robot using manufacturer’s recommendations (Perkin Elmer, Waltham, MA, United States). Completed libraries were quality checked and quantified using a combination of Qubit RNA HS (Thermo Fisher Scientific, Waltham, MA, United States) and Caliper LabChipGX HS RNA assays. All libraries were combined in equimolar amounts and pools were quantified using the Kapa Biosystems Illumina Library Quantification qPCR kit. Sequencing was performed in a single-end 50 bp read format using HiSeq 4000 SBS reagents and base calling was done by Illumina Real Time Analysis (RTA) v.2.7.6. Output of RTA was demultiplexed and converted to FastQ format with Illumina Bcl2fastq v2.19.0.

### Differential Gene Expression Analysis

Reads obtained from RNA sequencing were trimmed of adapter sequences and filtered to remove low-quality reads using Trimmomatic SE (Bolger et al., 2014) with the following parameters: -phred33 ILLUMINACLIP:2:30:10 LEADING:10 TRAILING:10 SLIDINGWINDOW:4:15 MINLEN:30. Trimmed and filtered reads were mapped to the *E. amylovora* ATCC49946 genome (Sebaihia et al., 2010) using bowtie2 (Langmead and Salzberg, 2012) with parameters: -q –phred33 -N 1. The resulting SAM file of mapped reads was sorted for downstream applications using SAMTools (Li et al., 2009). The *E. amylovora* ATCC49946 genome annotation file was used in conjunction with HTSeq (Anders et al., 2015) to count the number of reads mapping to each annotated feature. Read counts by feature across all samples were analyzed using the R package DESeq (Anders and Huber, 2012) to determine statistically differentially expressed genes between wild-type and Δ*arcZ* mutant samples with a false-detection rate of 0.05.

### Quantitative Real-Time PCR

For qRT-PCR validation of select differentially expressed genes, RNA samples were collected in the same manner as for RNA sequencing, from *E. amylovora* cultures grown in LB medium and then induced for 18 h in HIMM. Three replicate RNA isolations were made from independent bacterial cultures. 500 ng of total RNA was used as template for reverse transcriptase reactions using the High-Capacity Reverse Transcriptase kit (Applied Biosystems, Foster City, CA, United States) following prescribed protocols. Resulting cDNA was utilized as template in qRT-PCR reactions set up using SYBR green 2X master mix (Applied Biosystems, Foster City, CA, United States) according to manufacturer’s protocols and run on an Applied Biosystems StepOnePlus instrument. The housekeeping gene *recA* was included as an endogenous control, and relative mRNA abundance was calculated using the 2^–ddCt^ method (Livak and Schmittgen, 2001).

### Catalase Activity, Zone of Inhibition, and Minimum Inhibitory Concentration Assays

Catalase activity assays were conducted as described (Iwase et al., 2013), using cells grown overnight in liquid LB and adjusted to an OD_600 nm_ of 0.5. Briefly, cells were mixed in a 1:1:1 volumetric ratio with 8 M hydrogen peroxide and 1% (v/v) Triton X-100, allowed to incubate for 10 min and height of stabilized bubbles measured. These assays were carried out with 100 μL volumes in 15 mm tubes, and measurements were normalized to those of wild-type cells. Zone of inhibition was assayed by spread-plating 100 μL of bacteria cultures with an OD_600 nm_ of 0.2 onto agar plates and then placing a filter paper disk in the center of the plate. A total of 10 μL of 8 M H_2_O_2_ was dripped onto the filter paper, and plates were incubated for 24 h at 28°C, after which the plate was imaged and the area of the zone of clearing around the filter paper disk was quantified using ImageJ image analysis software (Abràmoff et al., 2004). For determination of the minimum inhibitory concentration (MIC) of H_2_O_2_, LB or minimal media (4 g L^–1^ L-asparagine, 2 g L^–1^ K_2_HPO_4_, 0.2 g L^–1^ MgSO_4_ 7H_2_O, 3 g L^–1^ NaCl, 0.2 g L^–1^ nicotinic acid, 0.2 g L^–1^ thiamin hydrochloride, 10 g L^–1^ sorbitol) were prepared with concentrations of hydrogen peroxide ranging from 0 to 20 mM. Cells were inoculated into these media at an initial density of 1 × 10^7^ cfu mL^–1^ and incubated with shaking at 28°C. The MIC was determined to be the concentration of hydrogen peroxide at which bacterial growth was completely inhibited by visual assessment after 24 h. Each of these assays was repeated at least four times on separate days with independent bacterial cultures.

### Survival in Tobacco Apoplast

The ability of bacterial cells to survive in the apoplast of *Nicotiana tabacum* leaves was assessed as described (Santander et al., 2018), with the modification that surviving bacterial populations were enumerated at 5 days post-inoculation by dilution plating, rather than across a time-course. Each strain was tested in at least three experiments with a total of at least six replicates.

### Quantitation of Hydrogen Peroxide in Apple Leaves

Hydrogen peroxide levels in apple leaves were determined using a potassium iodide method (Junglee et al., 2014). For the assay, apple leaves were inoculated as described (Koczan et al., 2009) with a cell suspension of wild-type *E. amylovora* cells at a density of 5 × 10^8^ cfu mL^–1^. Inoculated leaves were sampled at indicated time points and 1 cm diameter disks were punched from the leaves, homogenized in 1 mL reaction buffer (0.5 M potassium iodide, 0.025% trichloroacetic acid, 2.5 mM potassium phosphate, pH 7), and supernatants from homogenates were transferred to wells of a 96-well microtiter plate and incubated in the dark for 30 min. Following incubation, 345 nm absorbance was measured, and background color from leaf tissue was subtracted by using leaf disks punched from the same leaf, homogenized in reaction buffer in lacking potassium iodide. Absorbance values were converted to concentrations of hydrogen peroxide using a standard curve as described (Junglee et al., 2014). This experiment was repeated twice, and in each experiment at least two leaves were sampled at each time point and two pairs of punches were made from each leaf. In total, at least 12 replicate measurements were made at each time point.

### Swimming Motility

Swimming motility assays were conducted in soft-agar as described (Schachterle et al., 2019). Briefly, cells were grown overnight in LB, normalized to an OD_600 nm_ of 0.2, and stab inoculated in swimming motility medium (10 g L^–1^ tryptone, 5 g L^–1^ sodium chloride, 2.5 g L^–1^ agar). After 24 h of incubation at 28°C, plates were photographed and the area covered by swimming cells was quantified using ImageJ (Abràmoff et al., 2004). Four biological replicates were assessed.

### Immature Pear Virulence Assay

Virulence of strains was assessed in immature pears as described (Zhao et al., 2005). Briefly, immature pears were washed and sterilized using 10% bleach, after which they were wounded and inoculated with 10^3^–10^4^ cfu in a 1 μL droplet and incubated at 28°C under high humidity conditions. Inoculated pears were assessed every 24 h for water soaking or necrotic symptom development. Each strain was tested with eight biological replicates.

### Reporter Fusion Generation and Testing

For translational fusions, the 5’ untranslated region (UTR) of each gene of interest was amplified from the transcriptional start site through 20 amino acids into the coding region and cloned in-frame with *gfp* in plasmid pXG20 (Urban and Vogel, 2007) using an *in vivo* assembly approach (García-Nafría et al., 2016). For the *katA* promoter fusion, the 500 bases upstream from the *katA* start codon were amplified and cloned into plasmid pPROBE-NT (Miller et al., 2000). Strains harboring the reporter fusions were grown overnight in LB medium and assessed for GFP fluorescent output using a Tecan Spark plate reader (Tecan, Männedorf, Switzerland) with excitation wavelength of 488 nm and emission wavelength of 535 nm. Relative fluorescence was determined by normalizing arbitrary fluorescence units to cell density, and relative to the wild-type strain. Each strain was tested in at least four experiments.

### Regulon Analysis

Known *E. coli* transcription factor regulons were obtained from RegulonDB (Gama-Castro et al., 2010) and corresponding gene sequences were extracted from the *E. coli* K-12 genome (Blattner et al., 1997). *E. coli* gene sequences were used as queries to search for presence in *E. amylovora* using tblastx from BLAST+ (Camacho et al., 2009). If a BLAST hit had an *e*-value of less than 0.001, that gene from *E. coli* was considered present in *E. amylovora.* Using the assumption that if a transcription factor and its regulated genes are conserved across *E. coli* and *E. amylovora*, regulatory relationships are likely to be similar, we used this assessment to generate putative *E. amylovora* regulons for several transcription factors. Putative *E. amylovora* regulons were tested for significant overlap with the ArcZ regulon determined herein using Fisher’s exact test with adjustment for multiple hypothesis testing.

## Results

### Transcriptomic Characterization of the *E. amylovora* Δ*arcZ* Mutant Relative to Wild-Type

We sequenced the *E. amylovora* Ea1189 transcriptome using RNA from wild-type and Δ*arcZ* mutant cells induced for six or eighteen hrs in HIMM (Huynh et al., 1989). Our sequencing resulted in a total of 128.4 million reads generated, of which 96.9% had per-base quality scores greater than 30. Of these reads, 97.2 percent mapped to the *E. amylovora* ATCC49946 genome. Following normalization and statistical analysis, we found a total of 342 differentially expressed genes between wild-type and Δ*arcZ* mutant cells. Of these, 62 genes were differentially regulated after 6 h of induction (27 up-regulated, 35 down-regulated) and 302 were differentially expressed after 18 h of induction (176 up-regulated, 126 down-regulated) with 22 genes differentially expressed at both time points (19 down-regulated, 3 up-regulated). Principal component analysis, based on differentially expressed genes, showed that samples clustered by strain and time point (Supplementary Figure S1).

**FIGURE 1:**
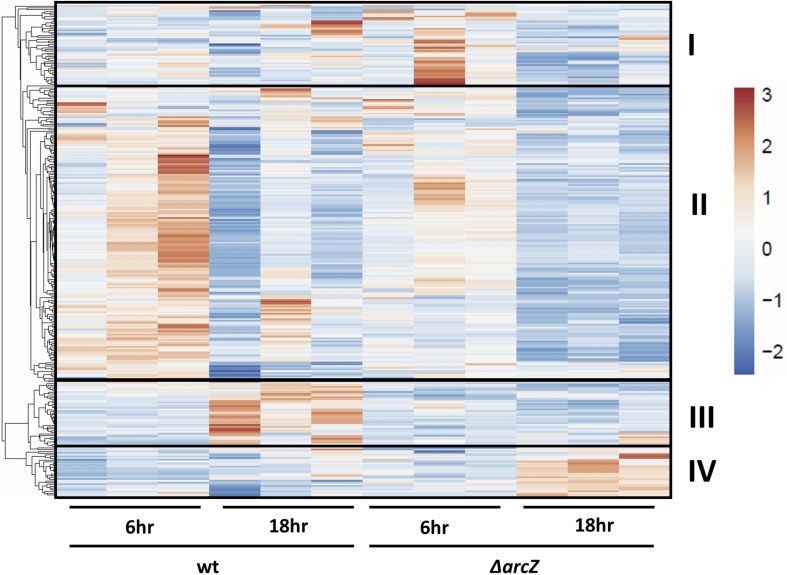
RNAseq heatmap comparing expression of differentially expressed genes across all biological replicate samples. Rows are centered and unit variance scaling is applied to rows. Rows are clustered using correlation distance and average linkage. Four main clusters of differentially expressed genes have been designated as groups I, II, III, and IV.

Visualization of differentially expressed genes across samples is provided as a heatmap in Figure 1. Genes clustered into four main groups by strain and time point differences, designated groups I, II, III, and IV. Group I genes are characterized by higher expression in the Δ*arcZ* mutant after 6 h of induction in HIMM, but no dramatic differences between wild-type and Δ*arcZ* at 18 h. Genes of interest in group I include the aerotaxis receptor, *aer*, and the leucine responsive regulatory protein, *lrp*, which we recently demonstrated is destabilized post-transcriptionally by ArcZ (Schachterle and Sundin, 2019). Group II genes are characterized by higher expression in wild-type samples at 6 h of induction relative to 18 h of induction in HIMM and reduced expression in general in the Δ*arcZ* mutant at both time points. This is the largest cluster of differentially expressed genes and includes genes involved in several metabolic and virulence processes. Examples of virulence associated genes include flagellar motility genes (*flhC*, *motB*, and *flgE*) and type III secretion genes (*hrpA*, *hrpW*, and *hrpJ*). Examples of metabolic genes include *crp* encoding the global regulator catabolite repressor protein, and other genes involved in metabolism such as *argD*, *cysD*, *gcvP*, *livM*, and *metB*. Group III genes are characterized by higher expression in wild-type at 18 h in HIMM compared to wild-type after 6 h of induction in HIMM, but not elevated in the Δ*arcZ* samples after 18 h of induction in HIMM. Many of these genes are also general metabolism genes and include *tktA* and *rpsS*. Group IV genes have elevated expression in the Δ*arcZ* mutant cells after 18 h of induction in HIMM. Most of these genes are uncharacterized, but multiple genes in this group are likely involved in reactions with phospho-sugars, such as *pgsA* and EAM_1622. For a complete list of all differentially expressed genes, see Supplementary Tables S2, S3. Because of the long duration of our time-course sampling, it is likely that many of the observed differentially expressed genes may be affected only indirectly by ArcZ. Because our approach did not distinguish between direct and indirect regulation by ArcZ, for the purposes of this study, we consider all of the observed differentially expressed genes to be a part of the ArcZ regulon.

### Pathway Enrichment in ArcZ Regulon

**FIGURE 2:**
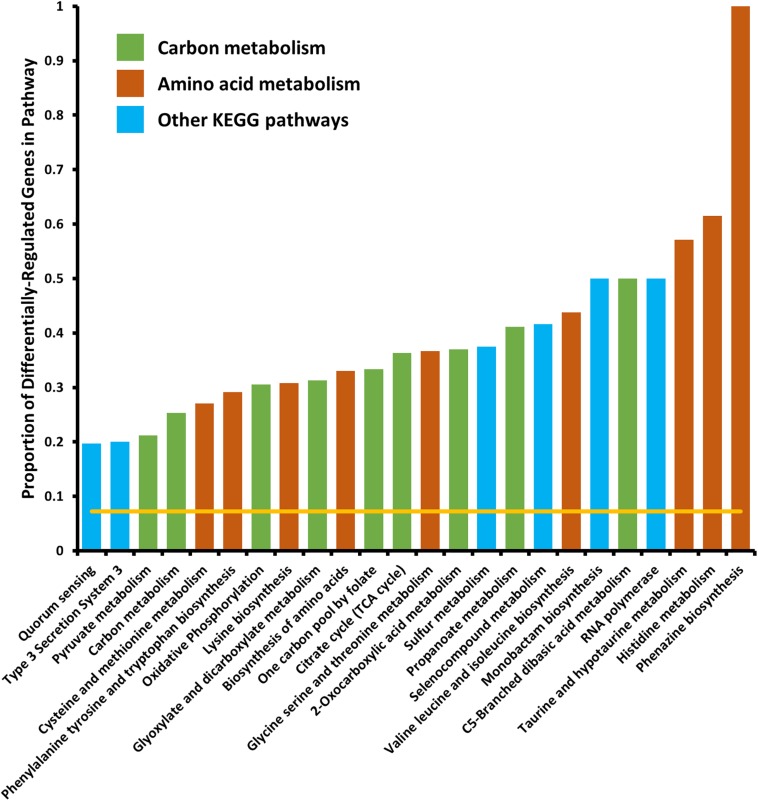
Kyoto encyclopedia of genes and genomes pathways significantly enriched in differentially expressed genes. Yellow line indicates expected proportion of differentially expressed genes if randomly distributed. Bars correspond to observed proportions of differentially expressed genes in KEGG pathways. Green bars indicate KEGG pathways related to carbon and central metabolism, burnt-orange bars indicate KEGG pathways relating to amino acid biosynthesis and metabolism and blue bars indicate other KEGG pathways. All bars shown are significantly enriched (*P_adj_* < 0.05) by Fisher’s exact test.

We tested for enrichment of specific cell pathways as annotated by the Kyoto Encyclopedia of Genes and Genomes (KEGG) (Kanehisa and Goto, 2000). We found no pathways significantly enriched in the set of genes differentially expressed in the 6 h time point, however, at the 18 h time point we found several pathways that were significantly enriched in differentially expressed genes (Figure 2). Several pathways that were enriched were involved in carbon metabolism and amino acid biosynthesis and metabolism. Because we observed that *crp* mRNA was affected by deletion of *arcZ*, it is possible that the carbon metabolism related pathway effects are due to this regulation, but it remains unknown if these are direct or indirect effects. The several genes and pathways involved in amino acid biosynthesis and metabolism are likely targets of the transcription factor Lrp, which is known to be regulated by ArcZ (Schachterle and Sundin, 2019) and which we found to be differentially regulated in the Δ*arcZ* mutant in our transcriptomic analysis. The type III secretion system was also significantly enriched for differentially expressed genes, the function of which is known to be affected by deletion of *arcZ* (Zeng and Sundin, 2014). Other affected KEGG pathways included sulfur metabolism, selenocompound metabolism, monobactam biosynthesis, RNA polymerase, and quorum sensing. Some of these pathways, although annotated in the KEGG database, may not be functional in *E. amylovora* as experimental evidence is lacking.

When analyzing the KEGG pathway for glycolate/glyoxylate metabolism, we found that *E. amylovora* has neither genes coding for enzymes that generate glycolate nor glyoxylate. In other organisms, glycolate oxidase, which converts glycolate to glyoxylate, generates hydrogen peroxide as a byproduct of this enzymatic reaction (Fahnenstich et al., 2008), and catalase is considered to be a part of this pathway for the detoxification of the peroxide. Although *E. amylovora* does not code for a glycolate oxidase enzyme, plants do, and have been shown to use this enzyme for generating hydrogen peroxide as a pathogen defense mechanism (Rojas and Mysore, 2012; Rojas et al., 2012). This led us to search for other genes differentially regulated by ArcZ that may play a role in coping with oxidative stress.

### ArcZ Regulates Oxidative Stress Response Genes

**FIGURE 3:**
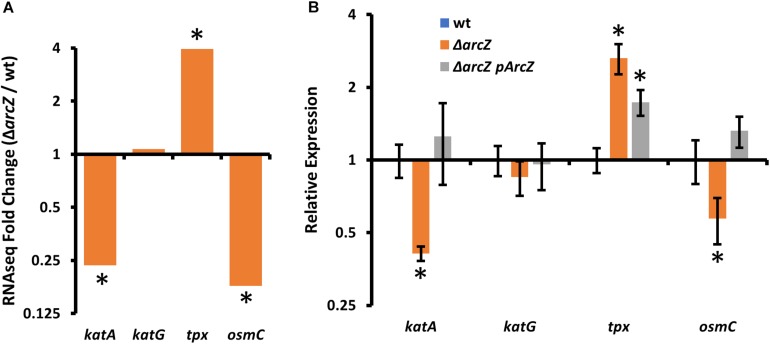
Oxidative stress mitigation enzymes are differentially expressed in *E. amylovora* Ea1189 Δ*arcZ* mutant relative to wild-type. Relative expression of *katA*, *katG*, *tpx*, and *osmC* based on **(A)** RNAseq (Δ*arcZ*/wt) or **(B)** quantitative real-time PCR. Asterisks indicate significant difference (*P* < 0.05) from wild-type by negative binomial distribution (RNAseq; through DESeq) or Student’s *t*-test (qPCR data). qPCR experiments used cDNA synthesized from three replicate RNA samples from independent bacterial cultures.

In our search of differentially expressed genes that have links to the oxidative stress response, we found *katA*, encoding a catalase, *tpx*, encoding a thiol-peroxidase, and *osmC*, encoding an osmotically inducible peroxiredoxin. *katA* and *osmC* were both down-regulated in the Δ*arcZ* mutant, and *tpx* mRNA was more abundant (Figure 3A). Although recent work has indicated that another catalase, KatG, plays a role in *E. amylovora* mitigation of oxidative stress (Santander et al., 2018), *katG* was not differentially expressed in the Δ*arcZ* mutant relative to wild-type. Nonetheless, as an additional oxidative stress mitigation enzyme, we have included *katG* in several of our experiments to better understand its role with the other ArcZ-regulated oxidative stress mitigation enzymes. We independently verified by quantitative real-time PCR that *katA* and *osmC* are down-regulated in the Δ*arcZ* mutant, and that *tpx* is up-regulated (Figure 3B). Consistent with our RNAseq data, there was no difference in relative abundance of *katG* mRNA between wild-type and the Δ*arcZ* mutant.

### ArcZ Regulated Oxidative Stress Response Genes Are Critical for Survival of Exogenous Hydrogen Peroxide

**FIGURE 4:**
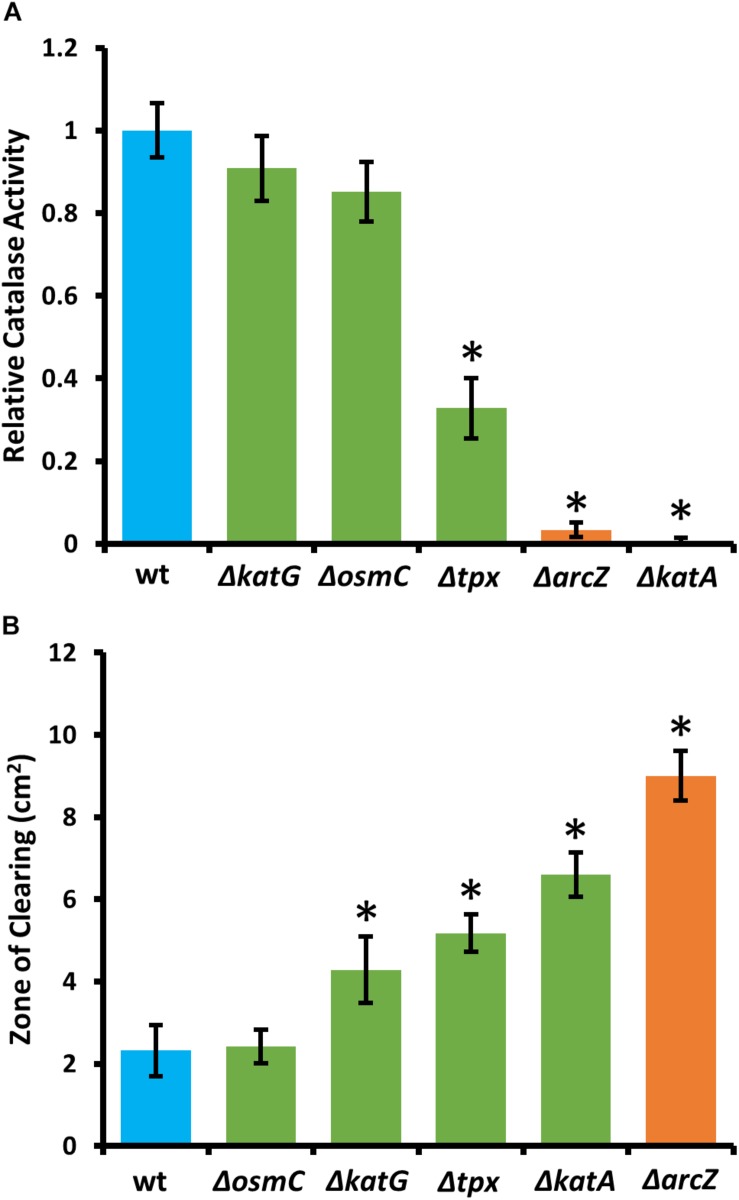
*E. amylovora* Ea1189 Δ*arcZ* mutant has reduced catalase activity and increased susceptibility to exogenous hydrogen peroxide. Relative catalase activity **(A)**, determined from cells grown overnight in LB broth. Zone of clearing **(B)** around a filter paper disk treated with 10 μL of 8 M hydrogen peroxide on LB solid media. Assays were repeated at least four times. Asterisks indicate significant (*P* < 0.05) difference from wild-type by Student’s *t*-test.

Because KatA and KatG have been shown to play a role in *E. amylovora* response to exogenous hydrogen peroxide (Santander et al., 2018), we tested the Δ*arcZ* mutant, along with the Δ*katA*, Δ*katG*, Δ*tpx*, and Δ*osmC* mutants for their catalase activity and survival after treatment with excess hydrogen peroxide. We found that the Δ*katA* mutant had no detectable catalase activity (Figure 4A) and exhibited increased susceptibility to hydrogen peroxide in a disk diffusion assay (Figure 4B). The catalase activity of the Δ*arcZ* mutant was reduced nearly 10-fold relative to wild-type and the mutant was also increased in sensitivity to hydrogen peroxide in a disk-diffusion assay. The Δ*tpx* mutant had a reduction in catalase activity of about 3-fold and increased sensitivity to hydrogen peroxide in the disk diffusion assay. The Δ*katG* and Δ*osmC* mutants had only a slight decrease in overall catalase activity, and the Δ*katG* mutant had increased susceptibility in the disk diffusion assay. It is likely that the Δ*katG* mutant did not show decreased catalase activity in the catalase activity assay but does have increased susceptibility in the disk-diffusion assay because of the differences in growth in liquid culture for the catalase activity assay and growth on solid media for the disk-diffusion assay, as it is known that *katG* expression is growth phase dependent (Santander et al., 2018). The growth of the Δ*osmC* mutant was not different from wild-type in the disk-diffusion assay.

During our determination of catalase activity in *E. amylovora*, we observed that a small amount of catalase activity is released into the culture medium. To determine whether the catalase activity in culture supernatants is from KatA or KatG, we concentrated culture supernatants from overnight cultures of the Δ*katA* and Δ*katG* mutants. Concentrated supernatants were mixed with hydrogen peroxide and monitored for evolution of gas through formation of bubbles. Catalase activity was observed in the Δ*katG* culture supernatants, but not in the Δ*katA* culture supernatants, indicating that KatA is responsible for the catalase activity present in culture supernatants. Because secreted catalase activity has not been reported in other *Enterobacterales*, we conducted a multiple sequence alignment of KatA and KatE protein sequences from phylogenetically diverse bacteria. This analysis revealed that *E. amylovora* KatA is more similar to KatA from *Bacillus subtilis* and *Pseudomonas aeruginosa* than to KatE from *E. coli* (Supplementary Figure S2). Protein BLAST (Camacho et al., 2009) further showed that the most similar hits for a search with *E. amylovora* KatA as query came from the genera *Erwinia*, *Pantoea*, and *Pseudomonas*.

### Mutation of ArcZ Can Be Complemented by *kat*A

**FIGURE 5:**
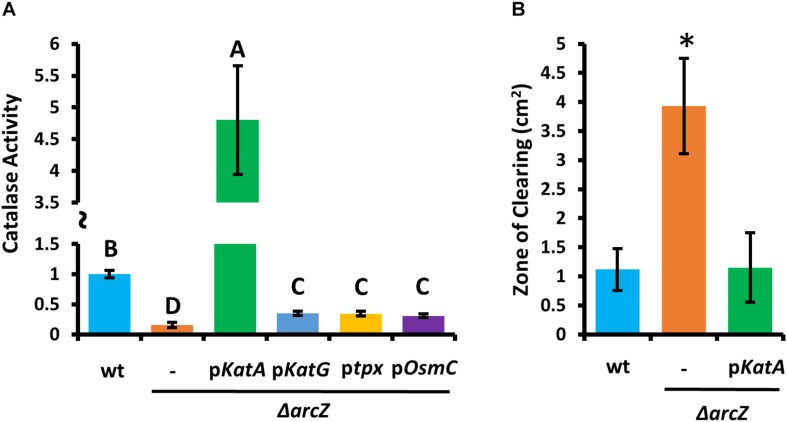
Providing *katA* on a plasmid recovers catalase activity and susceptibility to exogenous hydrogen peroxide in the *E. amylovora* Ea1189 Δ*arcZ* mutant. Relative catalase activity **(A)** of wild-type or Δ*arcZ* mutant carrying empty plasmid (-) or the indicated gene with its corresponding native promoter. Groups with shared letter designation do not differ from each other significantly (*P* < 0.05) by Tukey’s HSD test. Zone of clearing **(B)** around a filter paper disk treated with 10 μL of 1 M hydrogen peroxide on LB solid media. Tests were conducted at least four times and asterisks denote significant differences (*P* < 0.05) from wild-type by Student’s *t*-test.

Because Δ*arcZ* has reduced catalase activity relative to wild-type and is more susceptible than wild-type to exogenous hydrogen peroxide both on solid media and in liquid media, we wanted to determine if any of the oxidative stress mitigation enzymes would be able to restore wild-type phenotypes in these tests. To test this, we complemented the Δ*arcZ* mutant with *katA*, *katG*, *tpx*, or *osmC*, each with the respective native promoter in a medium-copy-number plasmid, where we hypothesized that increased gene copy number would compensate for moderate repressive regulatory effects. When tested for catalase activity, we found that introduction of any of these genes in this manner led to increased catalase activity relative to the Δ*arcZ* mutant (Figure 5A). However, providing *katG*, *tpx*, or *osmC* in the Δ*arcZ* mutant still resulted in catalase activity well below that of wild-type cells. Only providing *katA* on a plasmid restored catalase activity to greater than wild-type levels. When we tested the Δ*arcZ* mutant complemented with *katA* in the disk-diffusion assay for susceptibility to exogenous hydrogen peroxide, we found that *katA* restored wild-type levels of growth in the Δ*arcZ* mutant (Figure 5B).

### Hydrogen Peroxide Produced by Inoculated Apple Shoots

**FIGURE 6:**
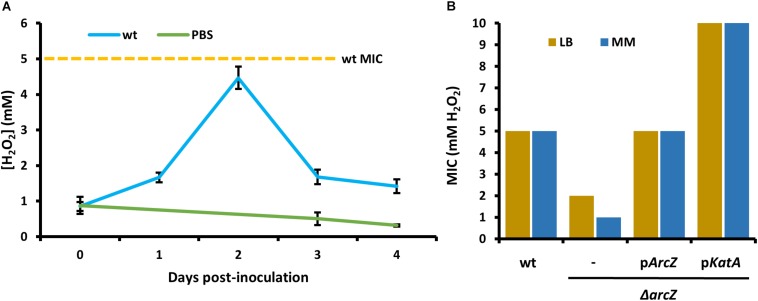
*Erwinia amylovora* Ea1189 elicits hydrogen peroxide production response from apple leaves and has evolved to cope with high levels of exogenous hydrogen peroxide. **(A)** Levels of hydrogen peroxide in apple leaves inoculated with wild-type *E. amylovora* or mock-inoculated with phosphate buffered saline (PBS), across time following inoculation. Averages come from two independent experiments with a total of at least 12 replicate measurements per time point, and error bars represent standard error of the mean. Dotted line indicates the H_2_O_2_ minimum inhibitory concentration (MIC) of wild-type *E. amylovora* Ea1189. **(B)** The MIC of hydrogen peroxide for wild-type or Δ*arcZ* mutant carrying empty plasmid (–) or the indicated gene with its native promoter. MIC was tested in LB and minimal media with sucrose as the carbon source (MM).

In order to relate the difference in hydrogen peroxide susceptibility of our various strains to the interactions between *E. amylovora* and host apple shoots, we quantified hydrogen peroxide levels in apple leaves over the course of infection with wild-type *E. amylovora* cells. We detected a baseline of approximately 1 mM hydrogen peroxide in uninfected apple leaves (Figure 6A). One day post-inoculation, before visual disease symptoms developed, hydrogen peroxide levels doubled to nearly 2 mM. After 2 days post-inoculation, when visual symptoms had developed in the main vein of the leaf, hydrogen peroxide levels had doubled again, to over 4 mM. After three- and 4-days post-inoculation, as visual fire blight symptoms spread from the main vein to the rest of the leaf, hydrogen peroxide levels decreased again to below 2 mM (Figure 6A).

In order to determine the specific concentration to which *E. amylovora* wild-type and Δ*arcZ* mutant cells are susceptible to exogenous hydrogen peroxide, we tested for the MIC of hydrogen peroxide. We found that the MIC of hydrogen peroxide for wild-type cells is 5 mM whether tested in minimal medium or rich LB medium (Figure 6B). The MIC of hydrogen peroxide for Δ*arcZ* mutant cells was found to be 1 mM in minimal medium and 2 mM when tested in LB medium. This is consistent with the finding that metabolism of specific amino acids available in rich media can help to mitigate oxidative threats (Carlioz and Touati, 1986) The hydrogen peroxide MIC of the Δ*arcZ* mutant was complemented back to wild-type levels by providing *arcZ* on a plasmid under control of its native promoter. The Δ*arcZ* mutant with *katA* on a plasmid grew uninhibited at concentrations of hydrogen peroxide up to 10 mM. It is noteworthy that the hydrogen peroxide MIC for wild-type cells was determined to be 5 mM, but *in planta* hydrogen peroxide levels peaked at just over 4 mM.

### ArcZ and KatA Are Critical for Survival of *E. amylovora* During the Hypersensitive Response in Tobacco

**FIGURE 7:**
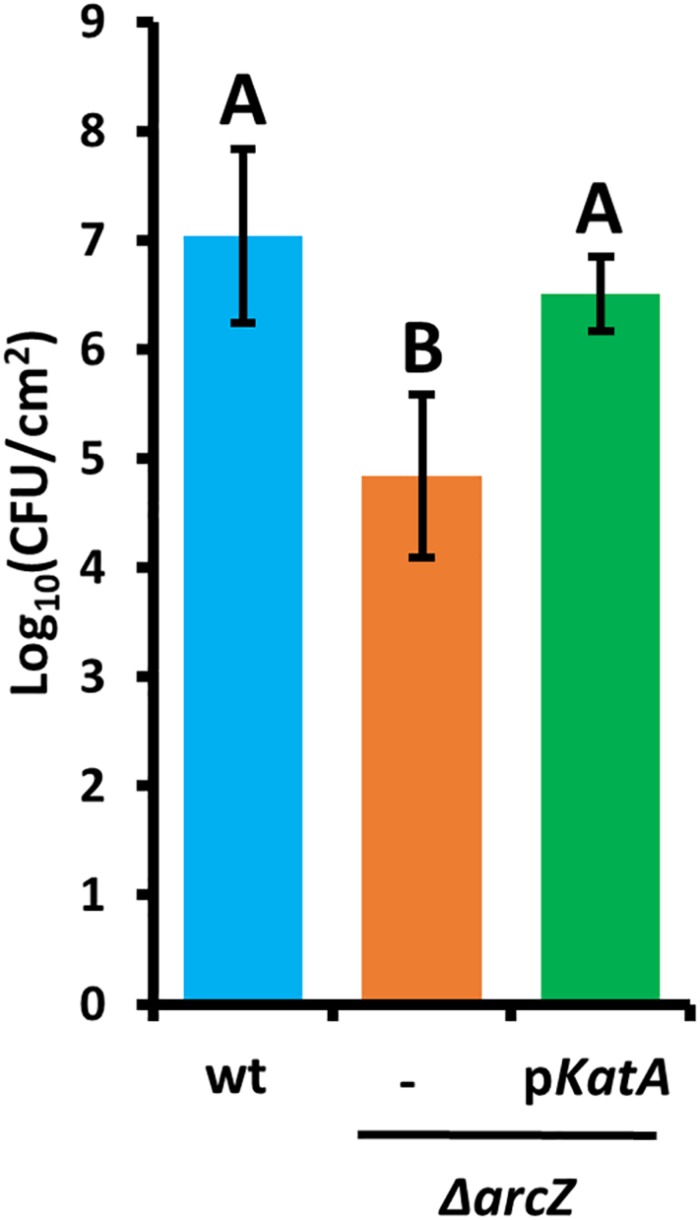
Survival of *E. amylovora* Ea1189 cells in tobacco leaves following elicitation of the hypersensitive response. Leaf disks were sampled 5 days post-infiltration. Each strain was tested at least three times with a total of at least six replicates. Error bars represent standard deviation and groups with shared letter designation do not differ significantly from each other (*P* < 0.05) by Tukey’s HSD test.

Because the hydrogen peroxide MIC for wild-type and Δ*arcZ* mutant cells and our quantification of hydrogen peroxide levels in apple leaves suggested that the inability of the Δ*arcZ* mutant to cope with oxidative stress may play an important role in ability of the bacteria to survive and successfully infect host plants, we wanted to test the impact of catalase activity on bacterial survival *in planta*. Because loss of *arcZ* leads to decreases in several virulence-associated traits, we also wanted to uncouple survival during the *in planta* oxidative burst from other virulence defects. To accomplish this, we assessed survival in non-host *Nicotiana tabacum* (tobacco) which will undergo a hypersensitive response, including an oxidative burst (Montillet et al., 2005), in response to type III effector translocation when *E. amylovora* cells are infiltrated into the tobacco apoplast (Wei et al., 1992). We infiltrated tobacco leaves with *E. amylovora* Ea1189 wild-type and Δ*arcZ* mutant cells at a density of 10^9^ CFU mL^–1^ and assessed survival 5 days post-infiltration by sampling a 1 cm^2^ leaf disk. We found that on average 10^7^ CFU/cm^2^ wild-type cells survived but only 10^5^ CFU/cm^2^ of Δ*arcZ* mutant cells survived (Figure 7). The survival defect in the Δ*arcZ* mutant could be rescued by providing *katA* on a plasmid, suggesting that the survival defect in the Δ*arcZ* mutant is due to increased susceptibility to reactive oxygen species, and not just to other pleiotropic effects of ArcZ. To verify whether provision of *katA* on a plasmid in the Δ*arcZ* mutant would be sufficient to complement the Δ*arcZ* virulence defect, we inoculated immature pears and monitored symptom development. We found that providing *katA* on a plasmid did not increase virulence of the Δ*arcZ* mutant in immature pears (data not shown).

### ArcZ Regulates *katA* Transcriptionally and *tpx* Post-transcriptionally

**FIGURE 8:**
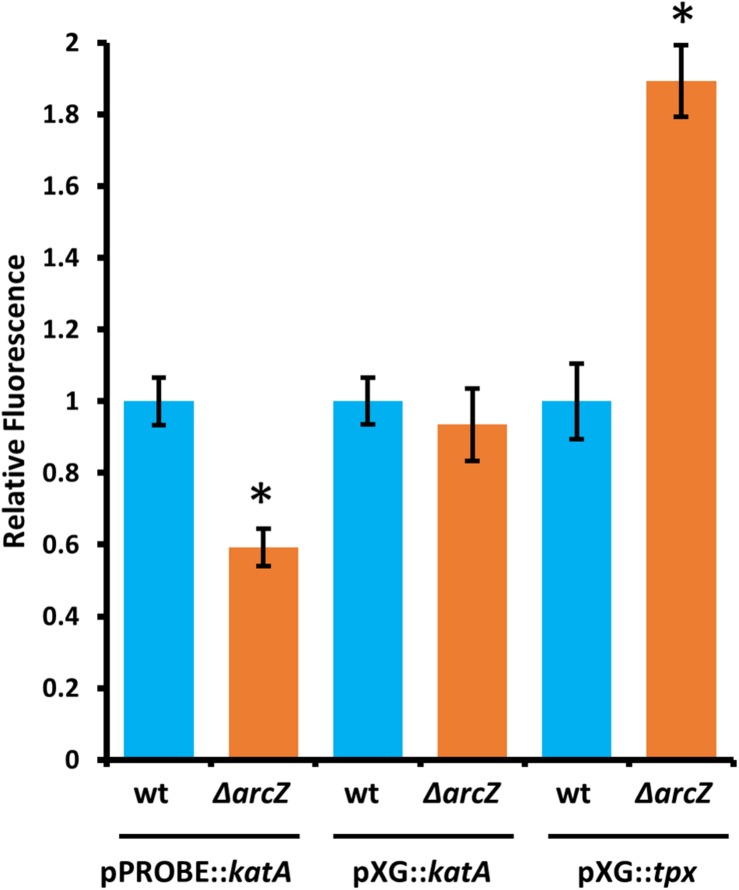
ArcZ of *E. amylovora* Ea1189 regulates *katA* promoter activity and regulates *tpx* post-transcriptionally. Relative fluorescence of indicated reporter fusions tested in Δ*arcZ* mutant cells and relative to fluorescence of wild-type cells carrying the same construct. Asterisks indicate significant difference (*P* < 0.05) by Student’s *t*-test between Δ*arcZ* and wild-type carrying the same construct. Error bars represent standard deviation, and each strain was tested at least four times.

Because ArcZ is a post-transcriptional regulator and modulates *katA* transcript abundance, we assessed whether ArcZ regulates *katA* at the transcriptional or post-transcriptional level. To do so, we constructed a promoter fusion with the *katA* promoter upstream of a promoter-less *gfp* in plasmid pPROBE-NT (Miller et al., 2000), and a translational fusion with the 5’ UTR of *katA* and first 18 amino acids in-frame with *gfp* in plasmid pXG20 (Urban and Vogel, 2007). We observed reduced *katA* promoter activity in the Δ*arcZ* mutant relative to wild-type but no difference on the *katA* translational fusion between wild-type and Δ*arcZ* (Figure 8).

Because ArcZ is known to post-transcriptionally repress *tpx* mRNA in *Salmonella enterica* serovar Typhimurium through a direct interaction (Papenfort et al., 2009), we tested whether ArcZ repression of *tpx* also occurs through post-transcriptional regulation in *E. amylovora*. We generated a translational fusion with the 5’ UTR of *tpx* and first 33 amino acids in-frame with *gfp* in plasmid pXG20 (Urban and Vogel, 2007), and compared relative fluorescence between wild-type and Δ*arcZ* mutant cells. We found increased GFP fluorescence in the Δ*arcZ* mutant relative to wild-type (Figure 8), suggesting that the ArcZ-*tpx* interaction is likely conserved between *Salmonella* Typhimurium and *E. amylovora*. To determine if this interaction is likely to occur between the same bases in these two organisms, we predicted the interaction between ArcZ and *tpx* using RNAhybrid (Krüger and Rehmsmeier, 2006), and found that the same region is predicted to interact in *E. amylovora* as in *Salmonella* Typhimurium (Supplementary Figure S3). Because the same interaction is predicted, and the fact that ArcZ has a high degree of conservation in the interacting region (Schachterle et al., 2019), it is likely that the post-transcriptional repression of *tpx* mRNA in *E. amylovora* occurs through the same interaction as in *Salmonella* Typhimurium. Because our qPCR analysis showed that *arcZ* complementation did not fully restore wild-type levels of *tpx* transcript, it is possible that in addition to post-transcriptional interactions, ArcZ may affect *tpx* indirectly at the transcriptional level.

### ArcZ Regulon Overlaps With Known Transcription Factor Regulons

**FIGURE 9:**
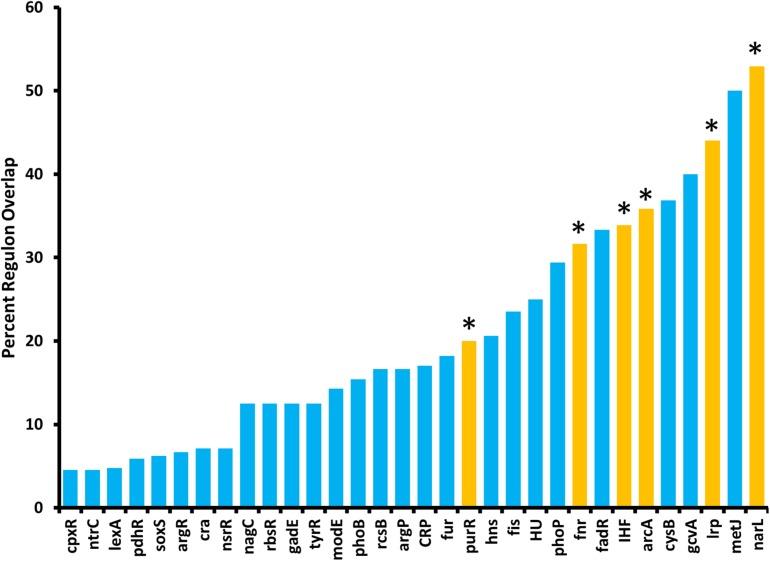
The *E. amylovora* Ea1189 ArcZ regulon overlaps with several putative transcription factor regulons. Transcription factor regulons were inferred in *E. amylovora* based on documented regulons in *Escherichia coli* and tested for significant (*P*_adj_ < 0.05; indicated by asterisks above bars) overlap with the ArcZ regulon by Fisher’s exact test. Transcription factor regulons with no overlap with the ArcZ regulon are not shown.

Because ArcZ regulates *katA* at the transcriptional level, we utilized our RNAseq data to search for candidate regulators that could explain the ArcZ regulation of *katA*. We analyzed the ArcZ regulon for overlap with known transcription factors with known regulons. We inferred *E. amylovora* transcription factor regulons on the assumption that if a transcription factor and its target gene are conserved between *E. coli* and *E. amylovora* then the target is also a part of the regulon in *E. amylovora*. We acquired *E. coli* regulon information from regulondb.com and utilized BLAST+ to search for transcription factor and target homologs in *E. amylovora*. Using this approach, we found 38 conserved regulators with conserved targets in those regulons, with an average of 48.5% of targets conserved in each regulon. When we tested these putative regulons for overlap with our determined *E. amylovora* ArcZ regulon, we found six regulons with a significant (*P*_adj_ < 0.05; Fisher’s exact test) amount of overlap (Figure 9). The six transcription factors with overlapping regulons are ArcA, Fnr, IHF, Lrp, NarL, and PurR. We note also that the overlap between the ArcZ and Fur regulons was nearly significant (*P*_adj_ = 0.069). Of these regulons, ArcA, Fnr and Fur all form a network of interactions and are known to have impacts on oxidative sensing and response (including catalase) in *E. coli* (Niederhoffer et al., 1990; Hassan and Sun, 1992; Compan and Touati, 1994; Benov and Sequeira, 2003). Furthermore, this core set ArcA, Fnr, and Fur also has known interactions with IHF (Mettert and Kiley, 2007), NarL (Tyson et al., 1993), and PurR (Stojiljkovic et al., 1994), three of the remaining regulators with ArcZ regulon overlap. Additionally, we recently reported that Lrp is regulated post-transcriptionally by ArcZ (Schachterle and Sundin, 2019).

### *arcZ* Mutant Phenotypes Are Recapitulated by *arcA* and *arcB* Mutants

**FIGURE 10:**
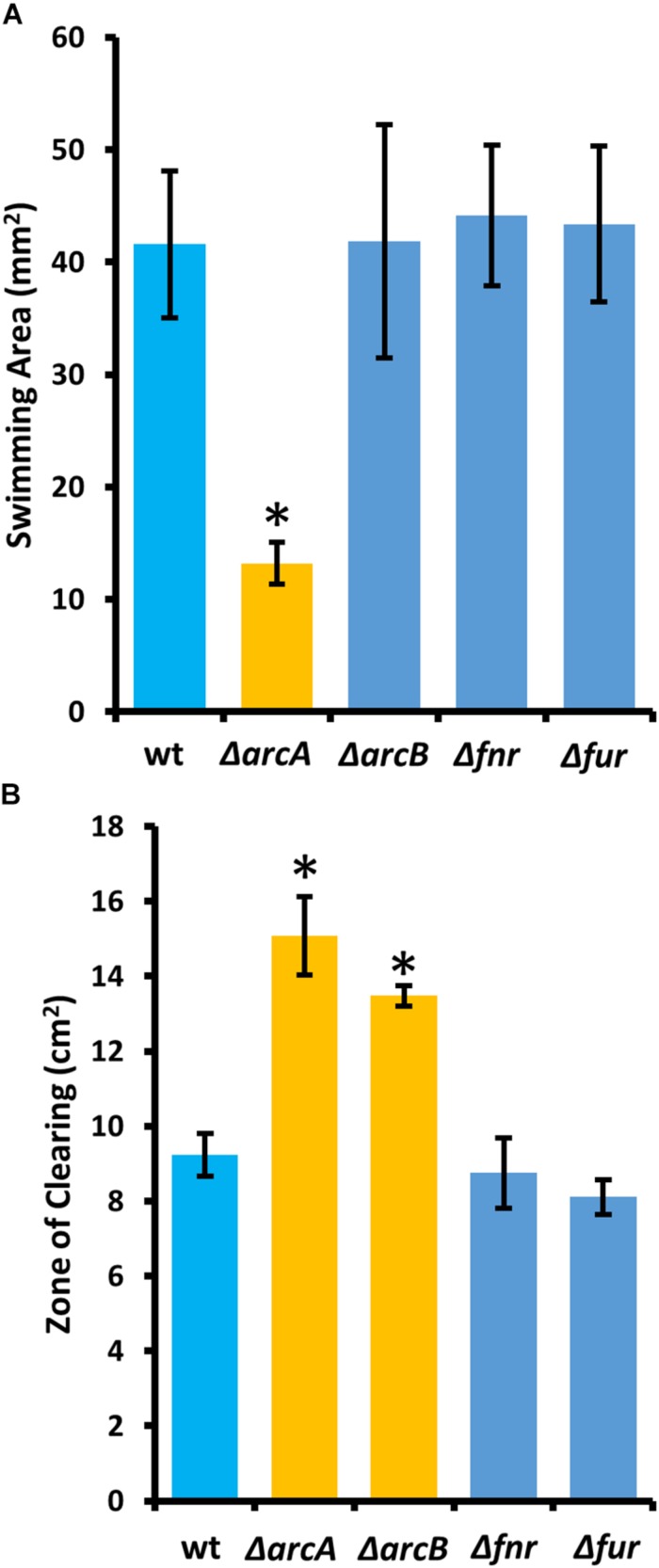
The *E. amylovora* Ea1189 ArcBA two-component system affects swimming motility and hydrogen peroxide susceptibility. **(A)** Swimming motility area of indicated strains 24 h after stab inoculation into soft agar (0.25% w/v) plates. **(B)** Zone of clearing around a filter paper disk treated with 8 M hydrogen peroxide on LB solid media. Asterisks indicate significant difference (*P* < 0.05) from wild-type strain by Student’s *t*-test. Error bars represent standard deviations from four biological replicates for swimming motility and from three biological replicates for hydrogen peroxide susceptibility.

To determine the regulatory roles that the ArcBA two-component system, along with Fnr and Fur may share with ArcZ, we generated single-gene deletion mutants for each of the genes encoding these transcriptional regulators. We determined the effect of these mutations on swimming motility and susceptibility to exogenous hydrogen peroxide, two phenotypic traits affected by deletion of *arcZ*. We found that the Δ*arcA* mutant had reduced swimming motility compared to wild-type, but that deletion of *arcB*, *fnr*, or *fur* had no effect (Figure 10A). Similarly, we found increased susceptibility to exogenous hydrogen peroxide in the Δ*arcA* and Δ*arcB* mutants compared to wild-type, but no difference in susceptibility to hydrogen peroxide in the Δ*fnr* and Δ*fur* mutants (Figure 10B).

### ArcZ Regulates ArcA Post-transcriptionally

**FIGURE 11:**
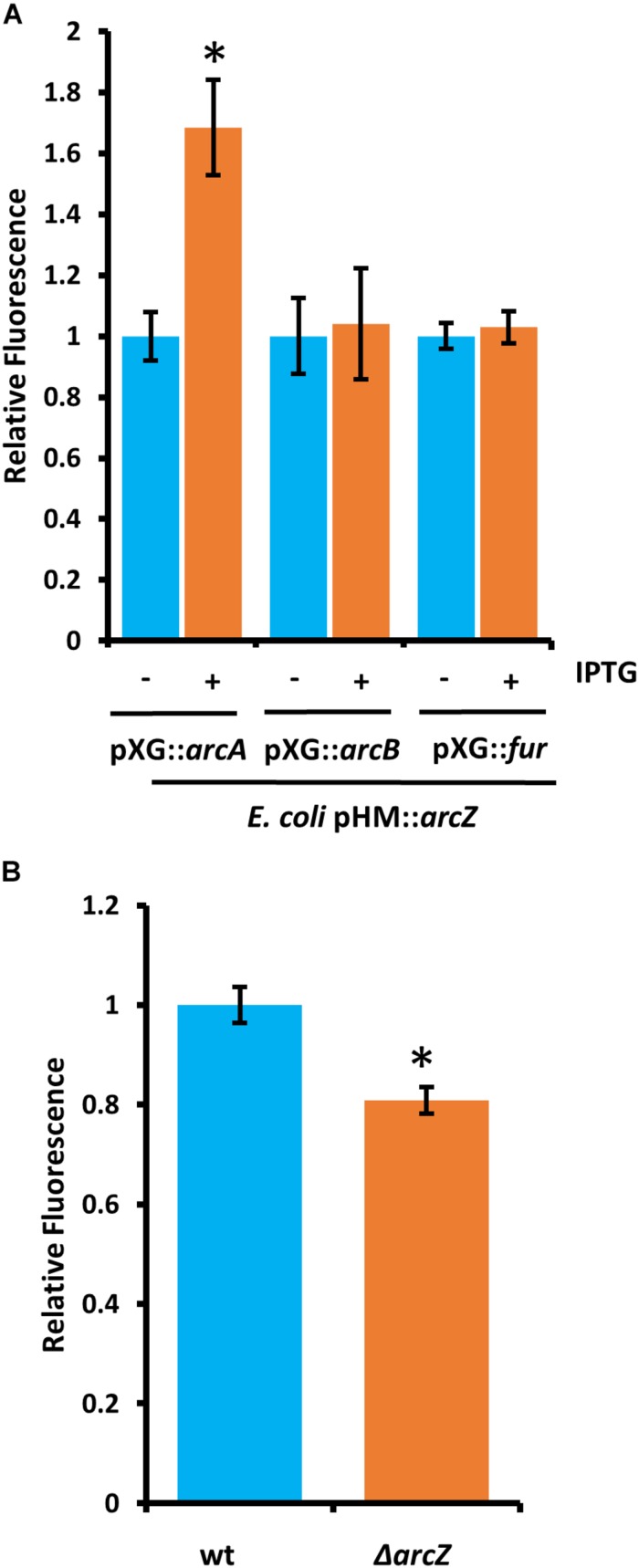
ArcZ of *E. amylovora* Ea1189 regulates *arcA* post-transcriptionally. **(A)** *Escherichia coli* carrying *E. amylovora arcZ* on a plasmid under control of the IPTG-inducible *tac* promoter, with the indicated translational fusions comparing relative fluorescence between un-induced and induced cells. **(B)** Relative fluorescence of *E. amylovora* wild-type or Δ*arcZ* mutant cells carrying the *arcA* translational fusion. Asterisks indicate significant difference (*P* < 0.05) from wild-type or un-induced cells carrying the same reporter plasmid by Student’s *t*-test. Error bars represent standard deviation from at least four biological replicates.

Because deletions in *arcA* or *arcB* of the ArcBA two-component system had similar effects to Δ*arcZ* on the motility and susceptibility to hydrogen peroxide phenotypes, we generated translational fusions for *arcA* and *arcB* to test whether ArcZ regulates these genes post-transcriptionally. We additionally generated a *fur* translational fusion to determine if ArcZ regulates *fur* post-transcriptionally because Fur is a transcriptional regulator of the catalase *katE* in *E. coli* (Benov and Sequeira, 2003). The *arcA*, *arcB*, and *fur* translational fusions with *gfp* reporter were tested in an *E. coli* strain carrying *arcZ* under control of an IPTG-inducible *tac* promoter. Upon induction of *arcZ* expression, we found no difference in the strain carrying the *arcB* or *fur* translational fusion constructs but did find increased fluorescence in the strain carrying the *arcA* construct (Figure 11A). To confirm this result, we tested the *arcA* translational fusion in *E. amylovora* wild-type and Δ*arcZ* mutant cells and found a 20 percent reduction in fluorescence in the Δ*arcZ* mutant compared to wild-type (Figure 11B). Together these results indicate that ArcZ regulates *arcA* post-transcriptionally in *E. amylovora*. We predicted candidate interactions between ArcZ and *arcA* mRNA using RNAHybrid (Krüger and Rehmsmeier, 2006) and found a strong candidate interaction 50 bases upstream of the ArcA start codon (Supplementary Figure S4). Consistent with the idea that ArcZ is affecting *katA* at the transcriptional level through post-transcriptional regulation of *arcA*, we found three direct repeats of the ArcA binding motif upstream of *katA* in the *E. amylovora* genome (Supplementary Figure S5). These three direct repeats of the ArcA binding motif represent a common arrangement of binding motifs in ArcA regulated genes (Park et al., 2013).

## Discussion

Here, we present transcriptomic analysis of the sRNA ArcZ regulon, providing evidence that in *E. amylovora*, ArcZ is a global regulator with a regulon of 342 genes, or 9.8% of the genome, based on the culture conditions used in our study. Furthermore, analysis of the ArcZ regulon identified an important role for ArcZ in regulation of genes involved in coping with oxidative stress. We found that ArcZ regulates *katA* at the transcriptional level and while it affects *tpx* transcript abundance, ArcZ represses *tpx* post-transcriptionally.

In addition to transcriptional regulation of *katA* and post-transcriptional regulation of *tpx*, we found that ArcZ regulates *arcA* post-transcriptionally. ArcA is the response regulator of the ArcBA (anoxic redox control) two-component system, which is responsive to oxidative status of the cell (Iuchi et al., 1990). This two-component system is activated in a sigmoidal response pattern in response to oxidative state of quinones (Bekker et al., 2010) and plays a major role in modulating expression of several genes encoding redox-active enzymes (Morales et al., 2013). The sRNA ArcZ has received this Arc acronym for its position adjacent to *arcB* in the genome as an arc-associated sRNA (Papenfort et al., 2009). Although *arcB* and *arcZ* are distal to *arcA* in the genome, it has been found in *E. coli* that *arcZ* is transcriptionally regulated in response to oxygen levels in an ArcA dependent manner (Mandin and Gottesman, 2010). Because we are reporting that ArcZ regulates *arcA* post-transcriptionally in *E. amylovora*, this suggests that if these regulatory relationships are conserved between *E. amylovora* and *E. coli*, ArcZ and ArcA may form a feedback loop to reinforce cellular responses in response to oxygen availability and oxidative status.

Given our findings that ArcZ regulates *katA* at the transcriptional level and *arcA* and *tpx* post-transcriptionally, we propose a regulatory model in which the ArcBA two-component system acts as an oxygen sensor to transcriptionally regulate *arcZ* and *katA*, and that ArcZ in turn activates *arcA* post-transcriptionally, providing positive feedback on catalase activity. ArcA regulates transcription of *arcZ* in *E. coli* in an oxygen dependent manner (Mandin and Gottesman, 2010), but further work is necessary to confirm that this same regulation occurs in *E. amylovora*. We hypothesize that this proposed regulatory loop is significant during infection of host tissue, because of variations in oxygen accessibility across tissues. For example, in tissues with high oxygen availability such as leaves and flowers, *E. amylovora* cells are interacting with living host cells that are the most prone to mount defense responses including production of reactive oxygen species. It has been shown previously that *E. amylovora* cells trigger host defense mechanisms including generation of an oxidative burst during compatible interactions (i.e., successful infection) (Venisse et al., 2002, 2003; Iakimova et al., 2013; Abdollahi et al., 2015). Indeed, we demonstrate here that concentrations of hydrogen peroxide in infected apple leaves peak at levels of 4–5 mM at 2 days post-inoculation (Figure 6A). In contrast, host cells are dead in mature xylem vessels, and host-produced reactive oxygen species are likely to be scarce. Furthermore, in woody xylem, it has been shown that oxygen levels are typically reduced to half of atmospheric oxygen with ample water flow, but that when xylem flow is restricted, oxygen levels can drop to anaerobic levels (Gansert, 2003). The oxygen-responsive nature of the proposed ArcZ-ArcA-KatA feedback loop suggests that oxygen and oxidative state may play an essential role in proper expression of genes for coping with reactive oxygen species during disease progression. Future work to determine the specific roles of oxygen availability as an environmental signal modulating virulence gene expression shows great promise to provide novel insights into how *E. amylovora* integrates environmental signals to determine virulence behaviors. Such insights are of great importance in understanding the basic biology of this pathogen to guide development of strategies that can limit its devastating effects.

In support of the importance of ability to cope with reactive oxygen species during infection, we found that provision of *katA* on a plasmid in the Δ*arcZ* mutant background not only restored catalase activity and wild-type susceptibility to exogenous hydrogen peroxide in *in vitro* tests, but also restored survival in non-host tobacco during hypersensitive response. This suggests that although Δ*arcZ* mutant cells are deficient in several virulence factors (Zeng and Sundin, 2014), coping with reactive oxygen species is a major limiting factor for this mutant *in planta* independent of other virulence-associated traits. This also matches the recent finding that the activity of the catalases KatA and KatG plays an important role in *E. amylovora* survival in the plant environment (Santander et al., 2018).

We found that ArcZ regulation of *katA* occurs at the transcriptional level and not at the post-transcriptional level. Previous research found little change in *katA* expression between exponential and stationary phase cultures (Santander et al., 2018), suggesting that ArcZ expression may not vary greatly either between these growth stages and that this regulatory system may respond to other environmental cues. We found that ArcZ regulates *arcA* and *tpx* post-transcriptionally and that interaction predictions between ArcZ and the *arcA* 5′ UTR indicate a likely interaction that could explain the effect of ArcZ on the *arcA* 5′ UTR, but further work is needed to provide experimental confirmation that these bases participate in direct interactions. The presence of three sequential ArcA binding sites upstream of *katA* suggests that the ArcZ regulation of *katA* is through the observed post-transcriptional effects on *arcA*. Again, future experimentation is necessary to confirm that ArcA directly regulates *katA* transcription.

The determined ArcZ regulon had significant overlap with the inferred regulons of ArcA, Fnr, PurR, Lrp, IHF, and NarL. Our previous work indicated that ArcZ regulates *lrp* (Schachterle and Sundin, 2019), and that finding was confirmed in this work in the significant amount of overlap between the ArcZ and Lrp regulons. In *E. coli*, the remaining transcription factors with regulon overlap with ArcZ form a complex web of inter-regulation, which is also involved in transcriptional regulation of catalases and thiol peroxidase (Niederhoffer et al., 1990; Hassan and Sun, 1992; Compan and Touati, 1994; Kim et al., 1999; Benov and Sequeira, 2003). The finding that ArcZ regulates *arcA* post-transcriptionally provides a connection between this sRNA and this transcription factor network, although additional links may exist. Although ArcZ affected abundance of *osmC* transcripts, deletion of *osmC* had little effect on the oxidative stress phenotypes we tested. Because *osmC* is a part of the *lrp* regulon (Bouvier et al., 1998), it seems likely that ArcZ is regulating *osmC* through its post-transcriptional regulation of *lrp* (Schachterle and Sundin, 2019). Because we found weak effects in the Δ*osmC* mutant when testing with hydrogen peroxide, it is possible that in *E. amylovora* as a peroxiredoxin OsmC functions to reduce the threat of organic peroxides but has little activity against inorganic hydrogen peroxide. Future work to understand the role of *osmC* and additional interactions between ArcZ and these transcription factors will help to uncover the contributions of these regulatory networks to *E. amylovora* physiology and virulence.

In this study, we observed catalase activity present in culture supernatants, and determined that *katA* is responsible for this activity. This suggests that during infection *E. amylovora* may be releasing or actively secreting catalase to reduce damage done to cellular structures when peroxide production is elicited as a part of host-defense responses. In support of the hypothesis that early protection may be important, production of hydrogen peroxide in infected apple and pear shoots occurs early during disease development, as elevated production of hydrogen peroxide occurred ahead of symptom development (Abdollahi et al., 2015). Additionally, because the protein sequence of *E. amylovora* KatA is more similar to catalases from gram-positive *Bacillus subtilis* than it is to KatE from *E. coli*, *E. amylovora* may have acquired this gene during its evolution as a plant pathogen. Indeed, KatA from *E. amylovora* is most similar to catalases from *Pantoea* and *Pseudomonas* species, suggesting it may have been horizontally acquired from one of these during evolution as bacteria from these genera all colonize apple flowers (Shade et al., 2013). Because *katA* does not encode a secretion signal peptide, further work will be needed to determine if KatA is being actively secreted or released through some other means such as simply as a result of cell lysis. Because we observed that KatA catalase activity is stable in the extracellular milieu, we hypothesize that extracellular KatA may play an important role during disease development.

In *E. amylovora*, ArcZ has been shown to directly interact with *flhDC* mRNA (Schachterle et al., 2019) and to post-transcriptionally regulate *lrp* (Schachterle and Sundin, 2019). In *Salmonella* Typhimurium, ArcZ is known to regulate and interact with *sdaCB*, *tpx*, and a gene encoding a horizontally acquired methyl-accepting chemotaxis protein (Papenfort et al., 2009). In *E. coli*, ArcZ is also known to interact with and post-transcriptionally regulate *rpoS* (Mandin and Gottesman, 2010). Herein we provide additional evidence that in *E. amylovora* ArcZ post-transcriptionally represses *tpx* similar to *S.* Typhimurium, and also acts as a post-transcriptional regulator of *arcA*. These interactions explain several of the phenotypes observed in the *E. amylovora ΔarcZ* mutant, however, additional phenotypes remain unexplained, such as the effects of *arcZ* on type III secretion. This transcriptomic and molecular analysis of the ArcZ regulon will serve to guide and inform future studies to more fully understand the mechanisms and specific roles that ArcZ plays as a global regulator in coordinating virulence-associated traits in *E. amylovora*.

## Data Availability Statement

The sequencing datasets generated in this study can be found in the NCBI Sequence Read Archive (SRA) under BioProject accession number: PRJNA543432 (https://www.ncbi.nlm.nih.gov/bioproject/PRJNA543432).

## Author Contributions

JS and GS conceived and designed the experiments. JS and DO conducted the experiments. JS, DO, and GS analyzed the data, and wrote and edited the manuscript.

## Conflict of Interest

The authors declare that the research was conducted in the absence of any commercial or financial relationships that could be construed as a potential conflict of interest.
