# Supplementary material for: Small RNA ArcZ Regulates Oxidative Stress Response Genes and Regulons in Erwinia amylovora
Source: Front Microbiol. 2019 Nov 29;10:2775. doi: 10.3389/fmicb.2019.02775 (PMC6895013; doi:10.3389/fmicb.2019.02775)
Supplement: Supplementary file 1 [file Data_Sheet_1.PDF]

Supplementary Material

to: ArcZ regulation of catalase in *E. amylovora*

by: Schachterle, J.K., Onsay, D.M., and Sundin, G.W.

Contents:

Table S1	p2
Table S2	p3
Table S3	p4
Figure S1	p9
Figure S2	p10
Figure S3	p11
Figure S4	p12
Figure S5	p13

Table S1: Oligonucleotides/primers used in this work

Identifier	Sequence	Use/Source
katA qPCR F	TGGACGCTTCACATGCAGAT	qRT-PCR gene specific
katA qPCR R	TGCGGCCAGACTTTAGTGAG	"
katG qPCR F	AACGTGGCGCTGGAAAATTC	"
katG qPCR R	CGTCAGCCACTCTTTCTCGT	"
tpx qPCR F	AAAGACTATGGCGTGGCGAT	"
tpx qPCR R	GCTGGCTATGAATGACCCGA	"
osmC qPCR F	TAAGCAGGGTAAAGGCACGG	"
osmC qPCR R	CAGCGCCGATCAACTCTTCC	"
katA Knockout F	GTACTACACTTATCGTCGAAAATAACCATTTTAACATGGAGAGTATAGCGGTGTAGGCTGGAGCTGCTTC	Site-directed deletion of *katA*
katA Knockout R	GGGTGCCGCATGCTCAAAAAAAAGCGCCTTGCAGGCGCTTTATTCTGAGGCATATGAATATCCTCCTTA	"
katG Knockout F	ATTGGCGACAGTTAAGCTGGCTTTGTCAATATGAGTGATGGAGTCCGAAAGTGTAGGCTGGAGCTGCTTC	Site-directed deletion of *katG*
katG Knockout R	CAGCCTTTAGCCAAATAAAAACCCGGTAAGTTATCCTTACCGGGTTTAGCCATATGAATATCCTCCTTA	"
tpx Knockout F	CGACCGACACTGAAAACGATAAATCATCATAAACAATAAAAGGATAGCTTGTGTAGGCTGGAGCTGCTTC	Site-directed deletion of *tpx*
tpx Knockout R	CGGCCAGCCAGCTAAGGCGCGCTCCAGACAAGGAGCGCACCACAGAAGAGCATATGAATATCCTCCTTA	"
osmC Knockout F	CAATACAGCGGCTATAATGGTAGCTGATGTTAAACAACCGGAGAACAACAGTGTAGGCTGGAGCTGCTTC	Site-directed deletion of *osmC*
osmC Knockout R	TCCTTAACAGTTTCCTGACTTAACCAGAACATCATCATCTTCAACCGGAGCATATGAATATCCTCCTTA	"
pBBR1 MCS F	CACTGCCCGCTTTCCAGTCGGG	(Schachterle et al., 2019)[1]
pBBR1 MCS R	CCATGCACCGCGACGCAAC	(Schachterle et al., 2019)[1]
katA complement F	CCCGACTGGAAAGCGGGCAGTGTGACCATCGCCTTCAGTTAC	*katA* complementation with native promoter
katA complement R	GTTGCGTCGCGGTGCATGGATTCAGCACTCAACAAAGGC	"
katG complement F	CCCGACTGGAAAGCGGGCAGTGGGGACTTGTTGCGGTTGACC	*katG* complementation with native promoter
katG complement R	GTTGCGTCGCGGTGCATGGGAGAGCTTTATGGATTCGCCG	"
tpx complement F	CCCGACTGGAAAGCGGGCAGTGCTCAATTCCTTAACGGGTTCG	*tpx* complementation with native promoter
tpx complement R	GTTGCGTCGCGGTGCATGGCTGCGTGAGTATGGCATCAG	"
osmC complement F	CCCGACTGGAAAGCGGGCAGTGGTCTCTCAAACCTTACGCCTG	*osmC* complementation with native promoter
osmC complement R	GTTGCGTCGCGGTGCATGGCCTAAAGCAGAAGGATTAGTGCG	"
pXG20 F	TGTGCTCAGTATCTCTATCACTGATAGGGATGTCAATCTC	(Schachterle et al., 2019)[1]
pXG20 R	GGTTCTGGCGAATTCATGAGCAAAGGAGAAGAACT	(Schachterle et al., 2019)[1]
pPROBE F	GAGGATCCCCGGGTACCGAGCTC	(Schachterle et al., 2019)[1]
pPROBE R	GCCGGCTTCCATTCAGGTCG	(Schachterle et al., 2019)[1]
katA promoter F	GAGCTCGGTACCCGGGGATCCTCCGCTATACTCTCCATGTTAAAATG	*katA* promoter fusion in pPROBE-NT
katA promoter R	GAGCTCGGTACCCGGGGATCCTCCGGACTCCATCACTCATATTGAC	"
katA UTR F	GAGATTGACATCCCTATCAGTGATAGAGATACTGAGCACAGTCGAAAATAACCATTTTAACATGG	*katA* translational fusion in pXG20 by in vivo assembly
katA UTR R	AGTTCTTCTCCTTTGCTCATGAATTCGCCAGAACCGACCGTTAAGGAATGCTCATC	"
tpx UTR F	GAGATTGACATCCCTATCAGTGATAGAGATACTGAGCACACGATAAATCATCATAAACAATAAAGG	*tpx* translational fusion in pXG20 by in vivo assembly
tpx UTR R	AGTTCTTCTCCTTTGCTCATGAATTCGCCAGAACCTTTGGCTACCAGAGTAAACGG	"
arcA Knockout F	AGCCGTATGTCCTGTTTCGATTTTTGTTGGCAATTTTAGGTAGCGATCACGTGTAGGCTGGAGCTGCTTC	Site-directed deletion of *arcA*
arcA Knockout R	GAGGTAAGCCGTGGGACGGGCAGCTCAACAGCGCCCGTCCCGCCGAGACATATGAATATCCTCCTTA	"
arcB Knockout F	TTTAAACAAATCCGGTATGATTGCGGCTATCAGGCTGAAAGGGACATTATGTGTAGGCTGGAGCTGCTTC	Site-directed deletion of *arcB*
arcB Knockout R	TCATTTTTTTTCAGCGTCTGTTACCCATTGCCGTAAAACTTCCATATCATCATATGAATATCCTCCTTA	"
fur Knockout F	GAGATTGACATCCCTATCAGTGATAGAGATACTGAGCACACATTGCGCTTTAGCGTCGAC	Site-directed deletion of *fur*
fur Knockout R	AGTTCTTCTCCTTTGCTCATGAATTCGCCAGAACCATGGCCTTCGGGTTCCTGAAG	"
fnr Knockout F	TAACTAAAATATGTAAATTAATGCGAGTCATTTATCATCGAGCGTAGATTGTGTAGGCTGGAGCTGCTTC	Site-directed deletion of *fnr*
fnr Knockout R	AAAAAGTGGTAAACGAATCAATCAACTAAAAATATCGATCCGGCCCGGTTCATATGAATATCCTCCTTA	"
arcA UTR F	GAGATTGACATCCCTATCAGTGATAGAGATACTGAGCACAGCATCATCTGGCACTAACCCAG	*arcA* translational fusion in pXG20 by in vivo assembly
arcA UTR R	AGTTCTTCTCCTTTGCTCATGAATTCGCCAGAACCAACCATGTAGCCTTCGGCTTC	"
arcB UTR F	GAGATTGACATCCCTATCAGTGATAGAGATACTGAGCACACCGGTATGATTGCGGCTATCA	*arcB* translational fusion in pXG20 by in vivo assembly
arcB UTR R	AGTTCTTCTCCTTTGCTCATGAATTCGCCAGAACCCGCTGAAGCCAGCAGCAACGA	"
fur UTR F	GAGATTGACATCCCTATCAGTGATAGAGATACTGAGCACACATTGCGCTTTAGCGTCGAC	*fur* translational fusion in pXG20 by in vivo assembly
fur UTR R	AGTTCTTCTCCTTTGCTCATGAATTCGCCAGAACCATGGCCTTCGGGTTCCTGAAG	"

[1]Schachterle, J.K, & Sundin, G.W. (2019) The leucine-responsive regulatory protein Lrp participates in virulence regulation downstream of small RNA ArcZ in *Erwinia amylovora*. *mBio*. doi:

Table S2: 6h wt vs Δ*arcZ* differentially-expressed genes

Gene Name	Locus Tag	Annotated function	Log_2 Fold Change	P_{adj}
sfsB	EAM_3090	sugar fermentation stimulation protein	4.27	2.4E-18
-	EAM_0763	CRISPR-associated protein	-3.65	5.9E-11
-	EAM_0764	CRISPR-associated protein	-3.57	1.8E-10
pqqA	EAM_0512A	coenzyme PQQ synthesis protein A	2.95	1.4E-09
-	EAM_0768	CRISPR-associated protein	-2.85	1.1E-08
-	EAM_1867	hypothetical protein	3.80	3.3E-08
prtE	EAM_3365	type I secretion system protein	-2.56	2.2E-07
-	EAM_2949	putative fimbrial protein	-2.52	2.8E-06
prtD	EAM_3366	type I secretion system protein	-2.32	3.3E-06
cas3	EAM_0762	CRISPR-associated helicase	-2.25	3.5E-06
-	EAM_0765	CRISPR-associated protein	-3.21	3.5E-06
-	EAM_0767	CRISPR-associated protein	-3.14	3.5E-06
-	EAM_2243	conserved hypothetical protein	-2.27	1.2E-05
-	EAM_0766	CRISPR-associated protein	-3.49	2.9E-05
-	EAM_0811	putative exported protein	2.10	3.1E-05
arnB	EAM_1090	UDP-4-amino-4-deoxy-l-arabinose--oxoglutarate	-2.16	3.1E-05
-	EAM_2103	glycosyl transferase	-2.01	8.1E-05
inh	EAM_3367	protease inhibitor	-2.33	1.4E-04
-	EAM_2953	putative short chain dehydrogenase	-1.92	2.6E-04
prtA	EAM_3368	zinc-binding metalloprotease	-3.29	7.2E-04
wbaP	EAM_1941	UDP-Gal::undecaprenolphosphate Gal-1-P	-1.80	1.2E-03
arnC	EAM_1091	undecaprenyl-phosphate	-1.91	1.3E-03
-	EAM_1868	hypothetical protein	2.62	1.3E-03
-	EAM_2602	hypothetical protein	1.80	1.3E-03
prtF	EAM_3364	type I secretion system protein	-2.62	1.8E-03
-	EAM_2973	non-ribosomal peptide synthetase	-1.70	2.3E-03
-	EAM_0769	CRISPR-associated protein	-1.73	2.5E-03
-	EAM_0740	outer membrane protease	1.64	3.2E-03
-	EAM_2376	two-component sensor kinase	1.80	3.5E-03
-	EAM_0927	putative phosphate starvation-inducible membrane	-1.62	4.3E-03
-	EAM_0052	putative reverse transcriptase (pseudogene)	-2.10	5.8E-03
-	EAM_0742	outer membrane protease	1.57	5.8E-03
-	EAM_1030	putative NUDIX-family hydrolase	1.67	9.7E-03
asr	EAM_1805	acid shock protein	2.92	9.8E-03
-	EAM_0290	putative DNA-damage-inducible protein	1.57	1.0E-02
-	EAM_0333	conserved hypothetical protein	1.57	1.2E-02
-	EAM_2134	conserved hypothetical protein	1.47	1.2E-02
-	EAM_3414	transcriptional regulator	-1.58	1.2E-02
-	EAM_3423	ABC transporter, substrate-binding protein	1.45	1.4E-02
-	EAM_3028	putative plasmid-related protein	1.55	1.6E-02
dapD	EAM_0816	2,3,4,5-tetrahydropyridine-2-carboxylate	1.42	1.8E-02
guaD	EAM_1716	guanine deaminase	1.48	1.9E-02
-	EAM_3039	conserved hypothetical protein	-1.39	2.0E-02
-	EAM_0491	conserved hypothetical protein	-1.40	2.1E-02
ltaE	EAM_1315	low specificity L-threonine aldolase	-1.40	2.4E-02
mltE	EAM_1530	membrane-bound lytic murein transglycosylase E	1.38	2.6E-02
pstC	EAM_3466	phosphate ABC transporter, permease protein	-1.41	2.8E-02
traF	EAM_0268	putative plasmid transfer protein	-1.38	3.1E-02
glnK	EAM_1004	nitrogen regulatory protein P-II	1.43	3.1E-02
-	EAM_2467	two-component sensor kinase	-1.33	3.1E-02
-	EAM_0739	putative glycosyl hydrolase	1.90	3.4E-02
-	EAM_1266	putative mechanosensitive ion channel protein	-1.32	3.4E-02
-	EAM_0492	putative membrane protein	-1.33	3.6E-02
glnA	EAM_0032	glutamine synthetase	1.35	3.6E-02
orn	EAM_0436	oligoribonuclease	1.39	3.7E-02
-	EAM_0965	putative lipoprotein	-1.53	3.7E-02
-	EAM_0741	putative glycosyl hydrolase	2.61	3.9E-02
-	EAM_0315	hypothetical protein	5.08	4.2E-02
lrp	EAM_1328	leucine-responsive AsnC-family transcriptional	1.49	4.4E-02
-	EAM_1392	putative membrane protein	-1.30	4.4E-02
pstS	EAM_3467	phosphate ABC transporter, substrate-binding	-1.29	4.8E-02
ubiF	EAM_1152	2-octaprenyl-3-methyl-6-methoxy-1,4-benzoquinol	1.64	4.9E-02

Table S3: 18h wt vs ΔarcZ differentially-expressed genes

Gene Name	Locus Tag	Annotated function	Log_2 Fold Change	P_{adj}
cmr	EAM_1997	multidrug translocase (chloramphenicol	4.08	2.8E-11
-	EAM_0927	putative phosphate starvation-inducible membrane	-2.72	2.6E-07
-	EAM_0494	putative exported protein	-2.58	2.7E-05
hrpA	EAM_2887	type III secretion system protein	3.66	2.7E-05
-	EAM_3425	ABC transporter, ATP-binding protein	2.63	2.7E-05
thrA	EAM_0642	bifunctional aspartokinase/homoserine	2.57	4.0E-05
thrC	EAM_0644	threonine synthase	2.53	4.0E-05
gcvP	EAM_2789	glycine dehydrogenase [decarboxylating] (glycine	2.47	4.6E-05
prtA	EAM_3368	zinc-binding metalloprotease	-3.91	5.6E-05
metE	EAM_0198	5-methyltetrahydropteroyltriglutamate--homocyst	2.21	8.9E-05
cas3	EAM_0762	CRISPR-associated helicase	-2.51	8.9E-05
osmC	EAM_1076	peroxiredoxin (osmotically-inducible protein C)	-2.48	8.9E-05
livG	EAM_3287	high-affinity branched-chain amino acid ABC	2.43	8.9E-05
-	EAM_3350	putative siderophore-interacting protein	2.80	8.9E-05
-	EAM_1077	putative membrane protein	-2.16	9.7E-05
sfsB	EAM_3090	sugar fermentation stimulation protein	2.48	1.2E-04
tktA	EAM_2814	transketolase 1	2.30	1.3E-04
-	EAM_0933	conserved hypothetical protein	-2.04	1.6E-04
livM	EAM_3288	high-affinity branched-chain amino acid ABC	2.38	1.7E-04
inh	EAM_3367	protease inhibitor	-2.70	1.9E-04
-	EAM_1414	hypothetical protein	-2.60	2.3E-04
katA	EAM_1736	catalase	-2.09	2.3E-04
-	EAM_0445	putative lipoprotein	-1.98	2.4E-04
-	EAM_2366	two-component sensor kinase	-2.06	2.4E-04
cysD	EAM_2690	sulfate adenylyltransferase subunit 2	2.12	2.4E-04
-	EAM_0579	conserved hypothetical protein	2.15	2.5E-04
-	EAM_1859	hypothetical protein	-2.41	2.5E-04
trpG	EAM_1878	anthranilate synthase component II (Glutamine	2.31	2.5E-04
-	EAM_2397	two-component sensor kinase	2.13	2.5E-04
cysJ	EAM_3112	putative sulfite reductase [NADPH] flavoprotein	2.24	2.5E-04
trpB	EAM_1881	tryptophan synthase beta chain	2.15	2.7E-04
-	EAM_0357	putative exported protein	-2.16	2.9E-04
-	EAM_1623	conserved hypothetical protein	-2.56	2.9E-04
livH	EAM_3289	high-affinity branched-chain amino acid ABC	2.20	2.9E-04
-	EAM_1532	transglycosylase-associated protein	-2.01	3.1E-04
cysG	EAM_2691	siroheme synthase [includes:	2.05	3.5E-04
-	EAM_2425	two-component sensor kinase	-2.21	3.7E-04
wbaP	EAM_1941	UDP-Gal::undecaprenolphosphate Gal-1-P	-2.07	3.8E-04
-	EAM_0252	putative transporter	-2.11	4.2E-04
cysN	EAM_2689	Sulfate adenylyltransferase subunit 1	1.94	4.2E-04
gltA	EAM_3111	putative glutamate synthase [NADPH] large	2.15	4.2E-04
pqqA	EAM_0512A	coenzyme PQQ synthesis protein A	1.96	4.2E-04
-	EAM_0909	conserved hypothetical protein	-2.12	4.4E-04
tdk	EAM_1914	Thymidine kinase	-2.82	4.4E-04
metB	EAM_0133	cystathionine gamma-synthase	2.09	4.5E-04
trpA	EAM_1882	tryptophan synthase alpha chain	2.03	4.6E-04
-	EAM_1674	conserved hypothetical protein	-2.14	5.1E-04
trpD	EAM_1879	anthranilate phosphoribosyltransferase	2.09	5.9E-04
ptsG	EAM_1466	glucose-specific PTS system, IIBC component	1.76	6.3E-04
sucD	EAM_1175	succinyl-CoA ligase [ADP-forming] subunit alpha	2.07	6.7E-04
araF	EAM_1698	L-arabinose ABC transporter, substrate-binding	1.72	6.7E-04
trpE	EAM_1877	anthranilate synthase component I	2.03	6.7E-04
metK	EAM_2826	S-adenosylmethionine synthetase	2.11	6.7E-04
araG	EAM_1699	L-arabinose ABC transporter, ATP-binding	1.79	6.9E-04
hrpN	EAM_2877	harpin	3.58	7.9E-04
-	EAM_3424	ABC transporter, permease protein	2.09	7.9E-04
tauC	EAM_3217	taurine ABC transporter, permease protein	2.14	8.4E-04
-	EAM_1481	hypothetical protein	-2.06	8.7E-04
-	EAM_0323	putative membrane protein	2.31	9.2E-04
-	EAM_0593	putative protein kinase	2.00	9.2E-04
-	EAM_2243	conserved hypothetical protein	-3.06	9.2E-04
metF	EAM_0135	5,10 methylenetetrahydrofolate reductase	1.90	1.1E-03

Table S3: 18h wt vs ΔarcZ differentially-expressed genes (continued)

Gene Name	Locus Tag	Annotated function	Log_2 Fold Change	P_{adj}
sucC	EAM_1174	succinyl-CoA synthetase beta chain	2.07	1.1E-03
-	EAM_1707	utative lipoprotein	-2.02	1.1E-03
livF	EAM_3286	high-affinity branched-chain amino acid ABC	2.08	1.1E-03
-	EAM_2948	putative fimbrial protein	-1.81	1.1E-03
tpx	EAM_1833	thiol peroxidase (scavengase P20)	1.99	1.3E-03
speC	EAM_2850	ornithine decarboxylase, constitutive	1.99	1.3E-03
thrB	EAM_0643	homoserine kinase	2.35	1.4E-03
-	EAM_0761	putative dioxygenase	-2.14	1.4E-03
hrpW	EAM_2873	putative pectate lyase	3.31	1.5E-03
-	EAM_0928	putative membrane-associated	-2.07	1.8E-03
leuA	EAM_0683	2-isopropylmalate synthase	1.92	2.0E-03
-	EAM_1801	putative integrase (partial)	-1.82	2.0E-03
-	EAM_0106	putative membrane protein	-1.69	2.3E-03
dcuR	EAM_1541	two-component response regulator	-1.88	2.3E-03
rplD	EAM_3197	50S ribosomal subunit protein L4	1.69	2.3E-03
-	EAM_3423	ABC transporter, substrate-binding protein	1.74	2.4E-03
coaA	EAM_0228	pantothenate kinase	1.84	2.4E-03
-	EAM_2073	hypothetical protein	-1.96	2.5E-03
coaA	EAM_0221	pantothenate kinase	1.83	2.5E-03
-	EAM_2076	putative membrane protein	-1.78	2.5E-03
oxaA	EAM_3457	inner membrane protein	1.85	2.5E-03
argE	EAM_0137	acetylornithine deacetylase	1.98	2.6E-03
-	EAM_0466	conserved hypothetical protein	-1.85	2.7E-03
tauB	EAM_3218	taurine ABC transporter, ATP-binding protein	2.04	2.7E-03
hisD	EAM_2142	histidinol dehydrogenase	1.81	3.1E-03
hisB	EAM_2144	histidine biosynthesis bifunctional protein	1.82	3.1E-03
mtnK	EAM_0889	methylthioribose kinase (methionine salvage	1.92	3.2E-03
-	EAM_0410	conserved hypothetical protein	-2.62	3.3E-03
-	EAM_0585	conserved hypothetical protein	1.82	3.3E-03
coaA	EAM_0229	pantothenate kinase	1.79	3.4E-03
-	EAM_0578	conserved hypothetical protein	1.84	3.4E-03
hisF	EAM_2147	imidazole glycerol phosphate synthase subunit	1.85	3.5E-03
livJ	EAM_3290	high-affinity branched-chain amino acid ABC	1.70	3.5E-03
ubiF	EAM_1156	2-octaprenyl-3-methyl-6-methoxy-1,4-benzoquinol	1.79	3.6E-03
-	EAM_2849	TetR-family transcriptional regulator	-1.79	3.7E-03
dppF	EAM_3390	dipeptide ABC transporter, ATP-binding protein	1.75	3.7E-03
-	EAM_0966	putative phospholipid-binding protein	-1.81	3.8E-03
trpC	EAM_1880	anthranilate isomerase	1.71	4.0E-03
-	EAM_1675	putative transposase (partial)	-2.60	4.3E-03
leuB	EAM_0682	3-isopropylmalate dehydrogenase	1.62	4.5E-03
-	EAM_3413	putative zinc-binding alcohol dehydrogenase	1.90	4.5E-03
araH	EAM_1700	L-arabinose ABC transporter, permease protein	1.56	4.5E-03
rpsR	EAM_0450	30s ribosomal subunit protein S18	1.85	4.5E-03
-	EAM_0580	conserved hypothetical protein	1.94	4.5E-03
-	EAM_3226	putative membrane protein	2.08	4.5E-03
-	EAM_0800	conserved hypothetical protein	-2.49	4.5E-03
ompF	EAM_1357	outer membrane porin	1.47	4.5E-03
tauA	EAM_3219	taurine ABC transporter, substrate-binding	1.92	4.7E-03
-	EAM_1319	hypothetical protein	-1.60	4.8E-03
-	EAM_2058	putative exported protein	1.73	5.0E-03
hisA	EAM_2146	1-(5-phosphoribosyl)-5-[(5-phosphoribosylamino)me	1.83	5.4E-03
-	EAM_1849	LysR-family transcriptional regulator	-1.64	6.2E-03
ompH	EAM_0828	periplasmic chaperone	1.71	6.2E-03
-	EAM_1011	conserved hypothetical protein	-1.72	6.4E-03
gcvT	EAM_2791	aminomethyltransferase (glycine cleavage system	1.53	6.4E-03
nuoG	EAM_2287	NADH dehydrogenase I chain G	1.68	6.7E-03
-	EAM_2852	integrase (partial)	-2.09	6.9E-03
purT	EAM_1994	phosphoribosylglycinamide formyltransferase 2	1.76	7.3E-03
hisH	EAM_2145	imidazole glycerol phosphate synthase subunit	1.85	7.3E-03
acs	EAM_0327	acetyl-coenzyme A synthetase	1.73	7.5E-03
-	EAM_0872	ABC transporter, substrate-binding protein	2.35	7.9E-03
rplC	EAM_3198	50S ribosomal subunit protein L3	1.61	7.9E-03

Table S3: 18h wt vs ΔarcZ differentially-expressed genes (continued)

Gene Name	Locus Tag	Annotated function	Log_2 Fold Change	P_{adj}
-	EAM_3266	hypothetical protein	-3.30	7.9E-03
-	EAM_0028	conserved hypothetical protein	-1.84	8.3E-03
-	EAM_2054	putative dehydrogenase (partial)	-1.67	8.7E-03
hisG	EAM_2141	ATP phosphoribosyltransferase	1.73	8.7E-03
hisI	EAM_2148	histidine biosynthesis bifunctional protein	1.74	8.7E-03
fhuA	EAM_0805	ferrichrome iron TonB-dependent receptor	1.65	8.8E-03
-	EAM_0907	hypothetical protein	-2.78	8.8E-03
icdA	EAM_1520	isocitrate dehydrogenase	1.57	8.8E-03
nuoL	EAM_2282	NADH dehydrogenase I chain L	1.72	8.8E-03
dppC	EAM_3392	dipeptide ABC transporter, permease protein	1.80	8.8E-03
rpsC	EAM_3192	30S ribosomal protein S3	1.56	9.2E-03
proS	EAM_0844	prolyl-tRNA synthetase	1.66	9.3E-03
cysC	EAM_2688	adenylyl-sulfate kinase	1.65	9.3E-03
carB	EAM_0661	carbamoyl-phosphate synthase large chain	1.65	9.9E-03
metR	EAM_0197	LysR-family transcriptional regulator	1.71	1.0E-02
hisC	EAM_2143	histidinol-phosphate aminotransferase	1.75	1.0E-02
rplP	EAM_3191	50S ribosomal subunit protein L16	1.48	1.0E-02
-	EAM_2103	glycosyl transferase	-2.08	1.0E-02
nuoK	EAM_2283	NADH dehydrogenase I chain K	1.85	1.0E-02
-	EAM_3407	putative transcriptional regulator (pseudogene)	-2.02	1.1E-02
argG	EAM_0139	argininosuccinate synthase	1.67	1.1E-02
cfa	EAM_1660	cyclopropane-fatty-acyl-phospholipid synthase	-1.53	1.1E-02
lpdA	EAM_0749	dihydrolipoyl dehydrogenase (E3 component of	1.52	1.1E-02
asnS	EAM_1358	asparaginyl-tRNA synthetase	1.61	1.1E-02
-	EAM_3311	putative signal transduction protein	-1.45	1.1E-02
-	EAM_0590	conserved hypothetical protein	1.66	1.2E-02
flhC	EAM_2033	flagellar transcriptional activator	-1.94	1.2E-02
-	EAM_0906	putative membrane protein	-1.54	1.2E-02
gcd	EAM_1088	quinoprotein glucose dehydrogenase	1.67	1.2E-02
ggt	EAM_3279	gamma-glutamyltranspeptidase	1.56	1.2E-02
-	EAM_0923	hypothetical protein	-1.47	1.2E-02
pgl	EAM_1206	6-phosphogluconolactonase	1.67	1.2E-02
-	EAM_2418	two-component sensor kinase	1.47	1.2E-02
hmuS	EAM_1642	hemin transport protein	1.92	1.2E-02
rplB	EAM_3195	50S ribosomal subunit protein L2	1.43	1.2E-02
-	EAM_2101	hypothetical protein	-1.50	1.2E-02
nuoI	EAM_2285	NADH dehydrogenase I chain I	1.68	1.2E-02
sucB	EAM_1173	dihydrolipoyllysine-residue succinyltransferase	1.53	1.2E-02
metL	EAM_0134	bifunctional aspartokinase/homoserine	1.54	1.3E-02
-	EAM_1266	putative mechanosensitive ion channel protein	-1.42	1.3E-02
-	EAM_2137	putative amino acid permease	1.62	1.3E-02
hrpF	EAM_2882	type III secretion system protein	3.02	1.3E-02
-	EAM_1287	ABC transporter, substrate-binding protein	1.44	1.3E-02
-	EAM_1264	radical SAM superfamily protein (pseudogene)	-3.69	1.4E-02
rplV	EAM_3193	50S ribosomal subunit protein L22	1.64	1.4E-02
-	EAM_2396	two-component sensor kinase	1.62	1.4E-02
dppD	EAM_3391	dipeptide ABC transporter, ATP-binding protein	1.71	1.4E-02
sucA	EAM_1172	2-oxoglutarate dehydrogenase E1 component	1.57	1.4E-02
nuoH	EAM_2286	NADH dehydrogenase I chain H	1.58	1.4E-02
-	EAM_0040	putative purine permease	1.53	1.4E-02
-	EAM_2944	putative exported protein	-1.53	1.4E-02
trxC	EAM_2607	thioredoxin 2	-1.51	1.4E-02

Table S3: 18h wt vs ΔarcZ differentially-expressed genes (continued)

Gene Name	Locus Tag	Annotated function	Log_2 Fold Change	P_{adj}
-	EAM_0887	methionine salvage pathway protein E-2/E-2'	1.68	1.5E-02
-	EAM_0269	hypothetical protein	-1.63	1.5E-02
rplS	EAM_2626	50S ribosomal protein L19	1.47	1.5E-02
-	EAM_0965	putative lipoprotein	-1.36	1.5E-02
-	EAM_2451	two-component sensor kinase	-1.50	1.5E-02
-	EAM_0710	type II secretion system lipoprotein	-1.61	1.5E-02
MasA	EAM_0886	methionine salvage pathway protein E-1	1.64	1.5E-02
metG	EAM_2199	methionyl-tRNA synthetase	1.51	1.5E-02
prtD	EAM_3366	type I secretion system protein	-1.50	1.5E-02
hldE	EAM_2994	bifunctional protein [includes: D-beta-D-heptose	1.61	1.6E-02
nuoN	EAM_2280	NADH dehydrogenase I chain N	1.53	1.6E-02
pgsA	EAM_1406	CDP-diacylglycerol--glycerol-3-phosphate	-1.42	1.7E-02
-	EAM_0587	putative exported protein	1.62	1.7E-02
nuoF	EAM_2288	NADH dehydrogenase I chain F	1.49	1.7E-02
pta	EAM_2302	phosphate acetyltransferase	1.52	1.7E-02
-	EAM_2463	two-component sensor kinase	-2.84	1.7E-02
traF	EAM_0268	putative plasmid transfer protein	-1.38	1.7E-02
priB	EAM_0449	primosomal replication protein N	1.55	1.8E-02
purH	EAM_0249	bifunctional purine biosynthesis protein PurH	1.49	1.8E-02
ilvA	EAM_0155	threonine dehydratase biosynthetic (threonin	1.56	1.9E-02
-	EAM_0763	CRISPR-associated protein	-1.57	1.9E-02
accC	EAM_3142	biotin carboxylase (acetyl-CoA carboxylase	1.52	1.9E-02
dppA	EAM_3394	dipeptide ABC transporter, substrate-binding	1.44	1.9E-02
ilvD	EAM_0154	dihydroxy-acid dehydratase	1.46	2.0E-02
-	EAM_0764	CRISPR-associated protein	-1.58	2.0E-02
-	EAM_2467	two-component sensor kinase	-1.27	2.0E-02
-	EAM_3377	putative exported protein	-1.32	2.1E-02
-	EAM_0266	conserved hypothetical protein	-3.38	2.2E-02
argD	EAM_3227	acetylornithine/succinyldiaminopimelate	1.57	2.2E-02
-	EAM_1480	conserved hypothetical protein	-2.27	2.2E-02
-	EAM_0351	conserved hypothetical protein	1.31	2.2E-02
hrcN	EAM_2898	type III secretion system protein	1.75	2.2E-02
arnB	EAM_1090	UDP-4-amino-4-deoxy-l-arabinose--oxoglutarate	-1.47	2.2E-02
-	EAM_2949	putative fimbrial protein	-2.14	2.2E-02
-	EAM_2953	putative short chain dehydrogenase	-1.36	2.2E-02
valS	EAM_3046	valyl-tRNA synthetase	1.50	2.2E-02
rpsQ	EAM_3189	30S ribosomal subunit protein S17	1.42	2.2E-02
-	EAM_0956	conserved hypothetical protein	-1.35	2.3E-02
atpC	EAM_3473	ATP synthase epsilon subunit	1.47	2.3E-02
-	EAM_0852	putative methyltransferase (pseudogene)	-1.34	2.3E-02
-	EAM_2592	conserved hypothetical protein	-1.34	2.3E-02
-	EAM_1399	hypothetical protein	-2.16	2.3E-02
-	EAM_0766	CRISPR-associated protein	-1.49	2.4E-02
-	EAM_1561	type III secretion system protein	-1.54	2.5E-02
-	EAM_0576	conserved hypothetical protein	1.69	2.5E-02
fabH	EAM_1456	3-oxoacyl-[acyl-carrier-protein] synthase III	1.62	2.6E-02
-	EAM_0863	putative DNA methylase	-1.52	2.6E-02
-	EAM_1086	putative lipoprotein	-1.39	2.6E-02
araC	EAM_1701	arabinose operon regulatory protein	1.30	2.6E-02
atpD	EAM_3474	ATP synthase beta subunit	1.44	2.6E-02
acnB	EAM_0772	aconitate hydratase 2 (citrate hydro-lyase 2)	1.31	2.6E-02
murI	EAM_0151	glutamate racemase	1.44	2.6E-02
gltP	EAM_0328	proton glutamate symport protein	1.56	2.6E-02
-	EAM_1312	putative lipoprotein	-1.44	2.6E-02
pqqD	EAM_0515	coenzyme PQQ synthesis protein D	-1.35	2.6E-02
yedP	EAM_1477	putative mannosyl-3-phosphoglycerate	-1.33	2.6E-02
rnc	EAM_2514	Ribonuclease III	-1.84	2.7E-02
-	EAM_2412	two-component sensor kinase	-1.35	2.7E-02
-	EAM_1384	putative exported protein	-1.87	2.7E-02
-	EAM_0799	Rhs-family protein	-2.23	2.7E-02
oppD	EAM_1894	oligopeptide transport ATP-binding protein	1.51	2.9E-02
-	EAM_0592	putative ATPase with chaperone activity	1.37	2.9E-02
-	EAM_2188	putative protease (pseudogene)	-1.43	2.9E-02
-	EAM_1403	conserved hypothetical protein	-1.45	3.0E-02
grxC	EAM_0094	glutaredoxin 3	1.57	3.0E-02
blc	EAM_0425	outer membrane lipoprotein	-1.38	3.0E-02

Table S3: 18h wt vs ΔarcZ differentially-expressed genes (continued)

Gene Name	Locus Tag	Annotated function	Log_2 Fold Change	P_{adj}
-	EAM_0888	putative translation initiation factor EIF-2B	1.42	3.0E-02
osmE	EAM_1616	osmotically inducible lipoprotein E	-1.71	3.2E-02
rlsC	EAM_0531	levan regulatory protein	-2.07	3.3E-02
purK	EAM_1059	phosphoribosylaminoimidazole carboxylase ATPase	1.37	3.3E-02
-	EAM_2435	two-component sensor kinase	-2.06	3.3E-02
rpsS	EAM_3194	30S ribosomal subunit protein S19	1.39	3.3E-02
-	EAM_0598	Rhs-family protein	1.58	3.3E-02
leuC	EAM_0681	3-isopropylmalate dehydratase large subunit	1.25	3.3E-02
clpX2	EAM_0993	ATP-dependent Clp protease ATP-binding subunit	-1.41	3.3E-02
glgB	EAM_3272	1,4-alpha-glucan branching enzyme	-1.76	3.3E-02
-	EAM_2369	two-component sensor kinase	-1.34	3.4E-02
-	EAM_1722	conserved hypothetical protein	-1.31	3.4E-02
-	EAM_0359	putative siderophore biosynthesis protein	1.56	3.4E-02
-	EAM_1618	putative sodium:dicarboxylate symporter	1.36	3.5E-02
-	EAM_2824	conserved hypothetical protein	-1.29	3.5E-02
-	EAM_2943	putative transcriptional regulator	-2.29	3.5E-02
-	EAM_2057	putative peroxidase	1.33	3.6E-02
-	EAM_0768	CRISPR-associated protein	-1.38	3.6E-02
mqo	EAM_0109	malate:quinone oxidoreductase	1.31	3.7E-02
ppc	EAM_0136	phosphoenolpyruvate carboxylase	1.24	3.7E-02
-	EAM_1622	putative phosphotransferase	-1.19	3.7E-02
uspB	EAM_3324	universal stress protein B	-1.33	3.7E-02
coaA	EAM_0222	pantothenate kinase	-1.30	4.0E-02
budB	EAM_0013	acetolactate synthase, catabolic	-1.45	4.0E-02
hflC	EAM_0439	protein HflC	1.43	4.0E-02
tauD	EAM_3216	alpha-ketoglutarate-dependent taurine	1.98	4.1E-02
-	EAM_2440	two-component sensor kinase	-1.45	4.1E-02
hrpI	EAM_2896	type III secretion system protein	1.92	4.1E-02
flgE	EAM_1442	flagellar hook protein FlgE	1.50	4.1E-02
motB	EAM_2031	chemotaxis protein	-1.59	4.1E-02
-	EAM_2472	two-component sensor kinase	1.35	4.2E-02
-	EAM_0864	putative type II restriction endonuclease	-1.25	4.3E-02
atpA	EAM_3476	ATP synthase alpha subunit	1.36	4.3E-02
-	EAM_0613	patatin-like phospholipase	-1.12	4.3E-02
-	EAM_1317	conserved hypothetical protein	-1.20	4.3E-02
-	EAM_2415	two-component sensor kinase	-1.30	4.4E-02
rpsJ	EAM_3199	30S ribosomal protein S10	1.32	4.4E-02
-	EAM_0557	major facilitator superfamily protein	-1.31	4.4E-02
-	EAM_2441	two-component sensor kinase	1.49	4.4E-02
-	EAM_1239	putative glycosyl transferase	-1.57	4.5E-02
crp	EAM_3225	catabolite gene activator (cAMP receptor	1.37	4.5E-02
fabD	EAM_1457	malonyl CoA-acyl carrier protein transacylase	1.37	4.5E-02
-	EAM_3161	ABC transporter, ATP-binding protein	-1.43	4.6E-02
fklB	EAM_0453	FkbP-type peptidyl-prolyl cis-trans isomerase	1.55	4.6E-02
-	EAM_2743	putative lipoprotein	-1.66	4.7E-02
gnd	EAM_2150	6-phosphogluconate dehydrogenase,	1.28	4.7E-02
hrpD	EAM_2884	type III secretion system protein	2.38	4.7E-02
-	EAM_1408	MarR-family transcriptional regulator	-1.52	4.8E-02
ampC	EAM_2914	beta-lactamase (cephalosporinase)	1.90	4.8E-02
-	EAM_1078	putative membrane protein	-1.99	4.8E-02
-	EAM_1097	conserved hypothetical protein	-1.75	4.8E-02
thrS	EAM_1627	threonyl-tRNA synthetase	-1.51	4.8E-02
-	EAM_2077	conserved hypothetical protein	-2.17	4.8E-02
nuoE	EAM_2289	NADH dehydrogenase I chain E	1.33	4.8E-02
aer	EAM_0329	aerotaxis receptor	-1.60	4.8E-02
-	EAM_0577	conserved hypothetical protein	1.34	4.8E-02
-	EAM_2774	hypothetical protein	-1.35	4.9E-02
ddc	EAM_2959	L-2,4-diaminobutyrate decarboxylase	1.34	4.9E-02
-	EAM_0589	conserved hypothetical protein	1.62	5.0E-02
-	EAM_2669	type III secretion system protein	-1.58	5.0E-02

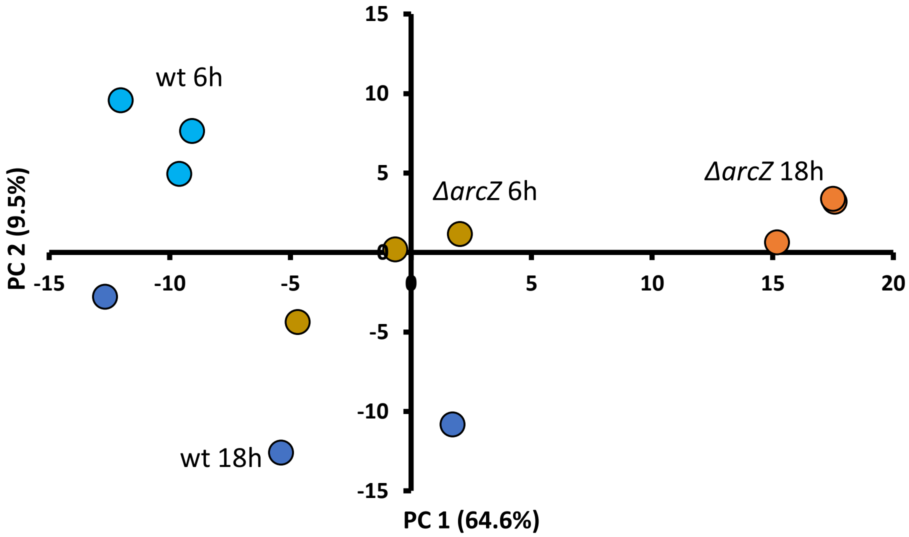

Figure S1 – Principal component analysis across all genes of wt and Δ*arcZ* RNAseq samples shows clustering by strain/timepoint.

A

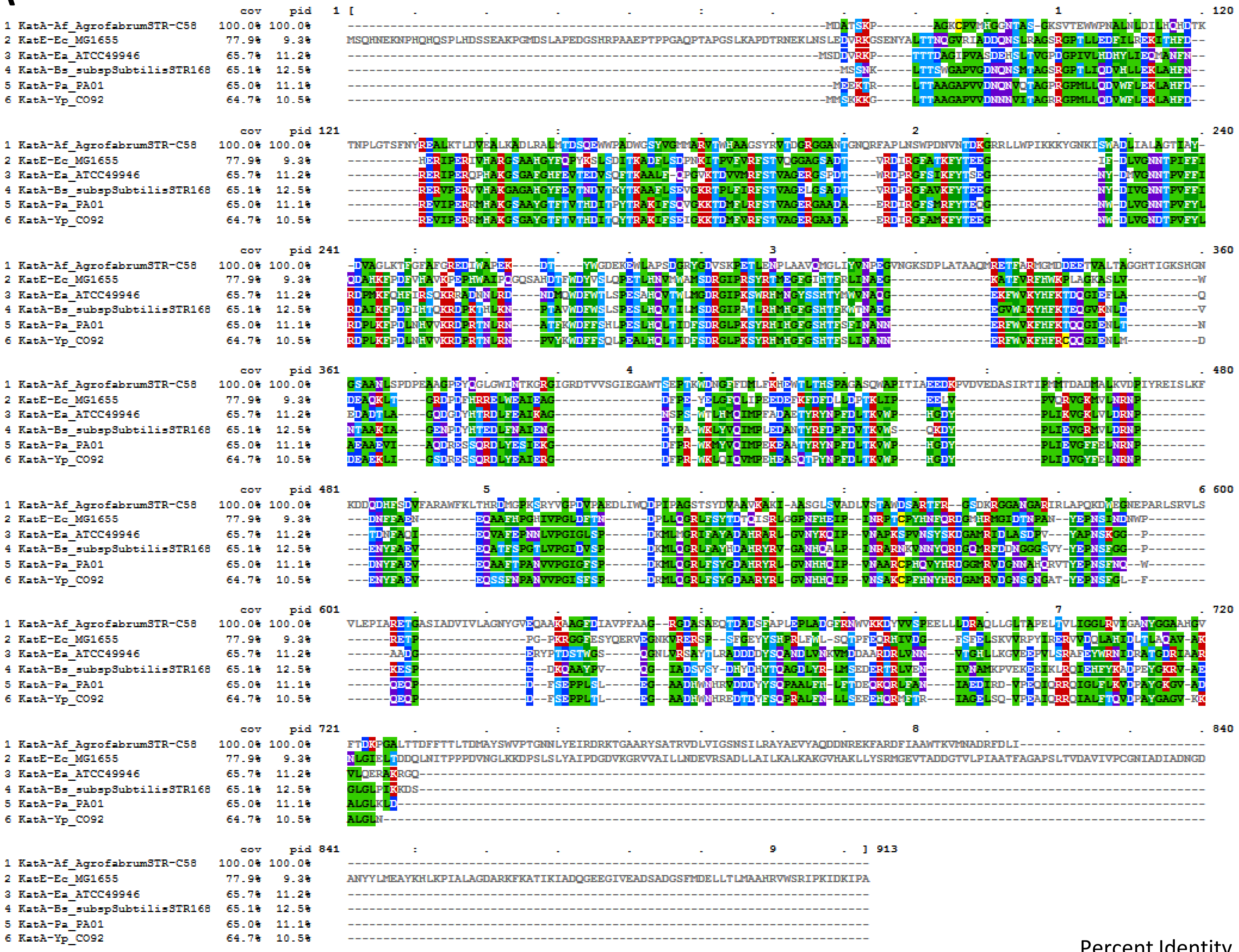

B

	KatA-A. fabrum STR-C58	KatE-E. coli MG1655	KatA-E. amylovora ATCC49946	KatA-B.subtilis STR168	KatA-P. aeruginosa PA01	KatA-Y. pestis CO92
KatA-A. fabrum STR-C58	100	15.1	15.37	17.2	15.32	14.53
KatE-E. coli MG1655	15.1	100	38.76	44.28	41.21	43.4
KatA-E. amylovora ATCC49946	15.37	38.76	100	51.04	48.54	47.38
KatA-B.subtilis STR168	17.2	44.28	51.04	100	55.11	53.88
KatA-P. aeruginosa PA01	15.32	41.21	48.54	55.11	100	80.17
KatA-Y. pestis CO92	14.53	43.4	47.38	53.88	80.17	100

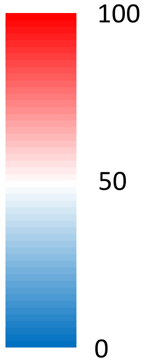

Figure S2 – KatA from *E. amylovora* is more similar to KatA from *Bacillus subtilis* and *Pseudomonas aeruginosa* than KatE from *Escherichia coli*. (A) Multiple sequence alignment of KatA and KatE protein sequences generated using MUSCLE and visualized using MVIEW. (B) Percent identity matrix based on multiple sequence alignment in (A).

```
mfe: -44.7 kcal/mol
p-value: 1.000000e+00

Position: 42
                                                     *
target 5' U       UAGCUU   U   A     UACAUCUUCA    AAUCC   CUC                  U             3'
           AAAAGGA      AUG CUC GACCG          GGGC     GGU   CGUGGA   UG GCCAGC GCCA
           UUUUUUU      UAC GGG CUGGC          UUCG     CCA   GCGCUU   AC CGGUUG UGGU
miRNA  3'                  U   G                   GCC     C        CUG  G           CCCUUUAA 5'
```

Figure S3 – ArcZ predicted interaction with *tpx* as predicted by RNAhybrid. Orange box indicates *tpx* start codon, green boxes indicate bases predicted to be involved in base-pairing in *Salmonella* Typhimurium, and asterisk indicates a base that reduced ArcZ-*tpx* interaction when mutated in *S.* Typhimurium.

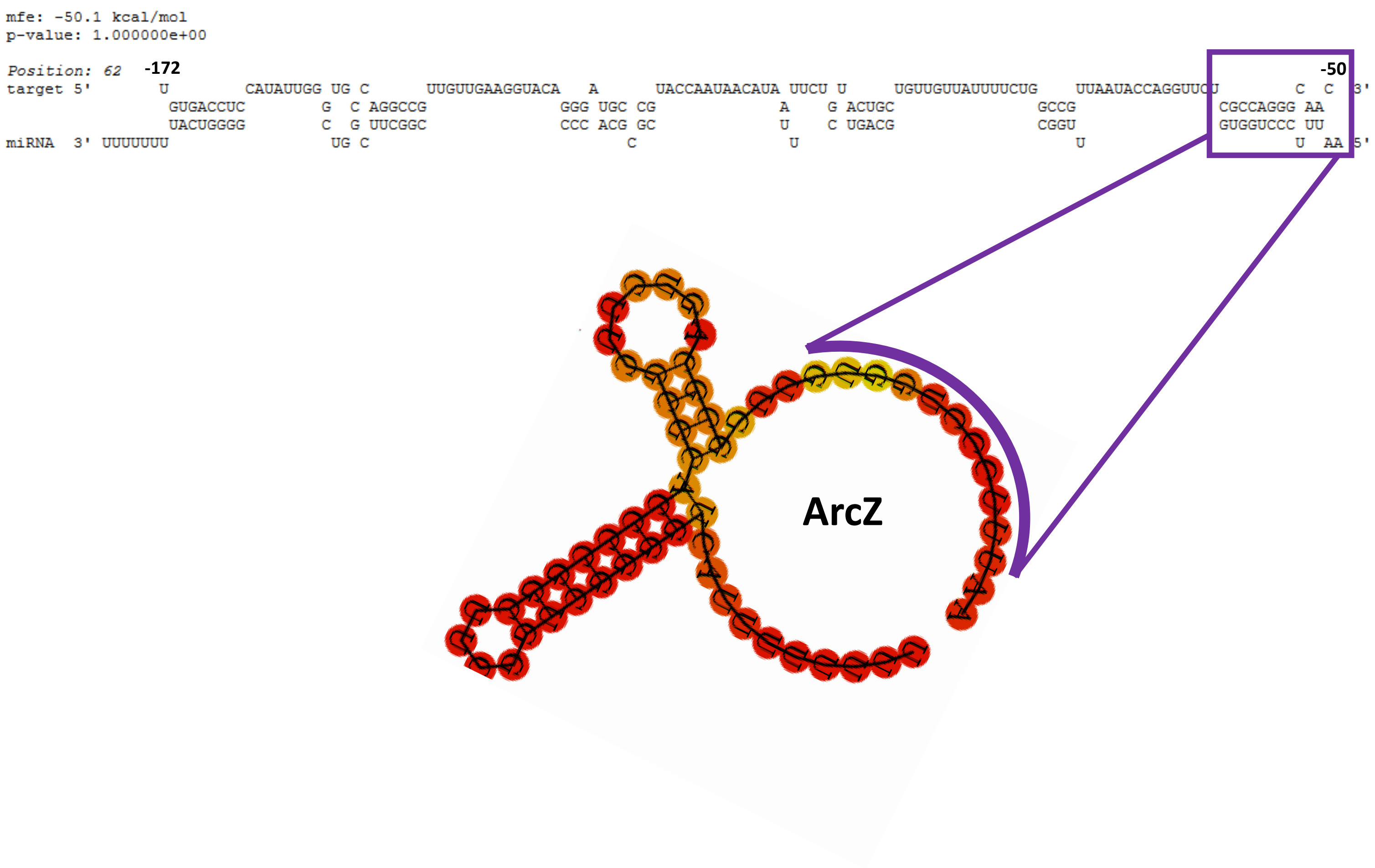

Figure S4 – ArcZ predicted interaction with *arcA* as predicted by RNAhybrid, with indication of accessible ArcZ bases typically involved in target interaction.

```
-192                                                                                                                                   -57
agtatctctccccttccagccagttccattgtgagaacaagcatcgccagctagccgtacggttgatgttatgccaaagctgcgattaatcttatttatccacatgctgtcaggtggataaaagg ccattgattc
                      ***   **** *                           **** *****          **                         *** * **             *** *
```

Figure S5 – ArcA predicted binding motifs upstream of *katA* in *E. amylovora* genome. The *katA* promoter has 3 ArcA motif direct repeats with good agreement with consensus motif at 21-22 bp intervals. Asterisks indicate bases that match the ArcA binding motif.
